# Alternative Oxidase Transcription Factors AOD2 and AOD5 of *Neurospora crassa* Control the Expression of Genes Involved in Energy Production and Metabolism

**DOI:** 10.1534/g3.116.035402

**Published:** 2016-12-16

**Authors:** Zhigang Qi, Kristina M. Smith, Erin L. Bredeweg, Natasa Bosnjak, Michael Freitag, Frank E. Nargang

**Affiliations:** * Department of Biological Sciences, University of Alberta, Edmonton, Alberta T6G 2E9, Canada; † Department of Biochemistry and Biophysics, Oregon State University, Corvallis, Oregon 97331-4003

**Keywords:** alternative oxidase, ChIP-seq, AOD2, AOD5, retrograde signaling

## Abstract

In *Neurospora crassa*, blocking the function of the standard mitochondrial electron transport chain results in the induction of an alternative oxidase (AOX). AOX transfers electrons directly from ubiquinol to molecular oxygen. AOX serves as a model of retrograde regulation since it is encoded by a nuclear gene that is regulated in response to signals from mitochondria. The *N. crassa* transcription factors AOD2 and AOD5 are necessary for the expression of the AOX gene. To gain insight into the mechanism by which these factors function, and to determine if they have roles in the expression of additional genes in *N. crassa*, we constructed strains expressing only tagged versions of the proteins. Cell fractionation experiments showed that both proteins are localized to the nucleus under both AOX inducing and noninducing conditions. Furthermore, chromatin immunoprecipitation and high throughput sequencing (ChIP-seq) analysis revealed that the proteins are bound to the promoter region of the AOX gene under both conditions. ChIP-seq also showed that the transcription factors bind to the upstream regions of a number of genes that are involved in energy production and metabolism. Dependence on AOD2 and AOD5 for the expression of several of these genes was verified by quantitative PCR. The majority of ChIP-seq peaks observed were enriched for both AOD2 and AOD5. However, we also observed occasional sites where one factor appeared to bind preferentially. The most striking of these was a conserved sequence that bound large amounts of AOD2 but little AOD5. This sequence was found within a 310 bp repeat unit that occurs at several locations in the genome.

Proper mitochondrial function requires coordinated expression between the mitochondrial and nuclear genomes. Although there is considerable variation in the coding capacity of mitochondrial DNA among different groups of eukaryotes (Bevan and Lang 2004; Burger *et al.* 2013), in all organisms the overall number of mitochondrial proteins encoded by the mitochondrial genome is many fold less than the number of mitochondrial proteins encoded by genes housed in the nucleus (Elstner *et al.* 2009; Meisinger *et al.* 2008). Thus, it is crucial that conditions affecting mitochondrial function or development be communicated to the nucleus so that expression of nuclear genes can be adjusted to meet the demands of changing conditions. The term retrograde regulation refers to changes in the expression of nuclear encoded genes in response to signals received from mitochondria (Liu and Butow 2006; Jazwinski 2013; da Cunha *et al.* 2015).

The standard electron transport chain (sETC) of mitochondria contains four large enzyme complexes as well as two smaller electron carriers, ubiquinone and cytochrome *c*, which shuttle electrons extracted from reduced electron carriers to molecular oxygen. Electron transfer is coupled to the pumping of protons across the mitochondrial inner membrane to establish a gradient that is harnessed by ATP synthase to make ATP (Lenaz and Genova 2009). Many organisms are able to produce an alternative oxidase (AOX) that provides a branch point from the sETC, which enables direct transfer of electrons from ubiquinol to oxygen, thus bypassing the later stages of the sETC (Moore *et al.* 2013). Genes encoding AOX exist in many species of plants, protists, fungi, primitive animals, and α-proteobacteria (McDonald *et al.* 2003, 2009; McDonald 2008; McDonald and Vanlerberghe 2006; Neimanis *et al.* 2013). In eukaryotes, AOX genes are housed in the nucleus. Thus, the protein is synthesized in the cytosol and imported into mitochondria where it localizes to the matrix side of the mitochondrial inner membrane.

In most fungi, AOX is undetectable, or present at very low levels, under standard laboratory growth conditions. However, inhibition of the sETC results in induction of AOX. This may occur via the action of chemical inhibitors, such as antimycin A or cyanide, which affect specific complexes of the sETC, or by mutations that decrease the function of sETC components (Tissieres *et al.* 1953; Edwards *et al.* 1976; Bertrand *et al.* 1983; Yukioka *et al.* 1998; Huh and Kang 2001; Dufour *et al.* 2000; Borghouts *et al.* 2001; Sakajo *et al.* 1993; Shi *et al.* 1999; Kirimura *et al.* 1996). Chemicals that specifically block mitochondrial protein synthesis, such as chloramphenicol (Cm), also induce AOX because they inhibit synthesis of mitochondrially encoded subunits of the sETC complexes (Tanton *et al.* 2003; Descheneau *et al.* 2005). Thus, AOX appears to provide an escape from conditions that block the function of the latter stages of the sETC. This allows continued ATP production via proton pumping at Complex I, and recycling of reduced electron carriers. Induction of AOX in response to a dysfunctional sETC represents a classic example of retrograde regulation.

Although the exact pathway and signals required for fungal AOX induction are not known, studies in *Neurospora crassa* (Chae *et al.* 2007b), *Podospora anserina* (Sellem *et al.* 2009), and *Aspergillus nidulans* (Suzuki *et al.* 2012) have shown that two zinc cluster transcription factors are required for the expression of AOX in response to sETC inhibition. The *N. crassa* proteins, called AOD2 and AOD5, are known to form a heterodimer that binds an alternative oxidase induction motif (AIM) consisting of two CGG triplets separated by seven nucleotides, found in the promoter region of the AOX-encoding *aod-1* gene (Chae *et al.* 2007a,b; Chae and Nargang 2009). In addition to their role in AOX expression, the orthologs of AOD2 and AOD5 in *P. anserina* (RSE2 and RSE3, respectively) and *A. nidulans* (AcuK and AcuM, respectively) are also known to be required for the expression of phosphoenolpyruvate carboxykinase (PEPCK) and fructose-1,6-bisphosphatase (FBP), two enzymes which are crucial for the process of gluconeogenesis (Sellem *et al.* 2009; Suzuki *et al.* 2012). These observations hint at a larger role for the transcription factors in cell growth and metabolism; indeed, a recent microarray study in *P. anserina* identified 598 genes whose expression is influenced by RSE2 and RSE3 (Bovier *et al.* 2014).

Here, we further define the mechanism of regulation that AOD2 and AOD5 play in *N. crassa*. The intracellular location of the transcription factors under conditions that do and do not induce transcription of AOX was examined. AOD2 and AOD5 were found to be constitutively localized to the nucleus. As in the other fungi previously examined, we found that the proteins are required for the expression of PEPCK, but we observed no effect on FBP expression. In addition, we conducted chromatin immunoprecipitation and high throughput sequencing (ChIP-seq), which showed that the proteins bind to the promoters of their target genes in both inducing or noninducing growth conditions. The ChIP-seq study also showed a wider involvement of the factors in controlling aspects of cell metabolism outside of AOX and gluconeogenesis.

## Materials and Methods

### Strains and growth of N. crassa

General handling and growth of *N. crassa* strains was as described (Davis and De Serres 1970). Unless otherwise specified, cells were grown using Vogel’s medium (Davis and De Serres 1970; Metzenberg 2003) with 44 mM sucrose as the carbon source. Experiments to measure growth rate and transcript levels in different carbon sources were done using synthetic crossing medium, which contains less available nitrogen (10 mM nitrate) compared with Vogel’s medium (25 mM nitrate and 25 mM ammonium). Carbon sources used were 44 mM sucrose, 217 mM glycerol, 217 mM ethanol, or 150 mM sodium acetate. When indicated, cells were grown in the presence of Cm at a final concentration of 2 mg/ml. Growth in the presence of Cm was used to achieve inducing conditions for expression of AOX. Unless stated otherwise, cultures were grown for 18 hr (in the absence of Cm), or 20 hr (in the presence of Cm) at 30°. Strains used in this study are described in Table 1.

**Table 1 N.** *crassa* strains used in this study

Strain	Genotype	Origin or Reference
NCN251	A	Fungal Genetic Stock Center (FGSC) # 987 (74-OR23-1A)
CNA33	*aod-2*, *pan-2*, a	Nargang laboratory (Chae *et al.* 2007b)
PL23-40	*aod-5*, *pan-2*, A	Nargang laboratory (Descheneau *et al.* 2005)
AOD2-N-HA-10	*aod-2*, *pan-2*, a	This study. Transformation of CNA33 with a hygromycin resistance plasmid carrying *aod-2* with triple HA tag coding sequence inserted after start codon
	Contains an ectopic copy of *aod-2* with N-terminal triple HA tag. Hygromycin resistant	
AOD2-C-HA-8	*aod-2*, *pan-2*, a	This study. Transformation of CNA33 with a hygromycin resistance plasmid carrying *aod-2* with triple HA tag coding sequence inserted before stop codon
	Contains an ectopic copy of *aod-2* with C-terminal triple HA tag. Hygromycin resistant	
AOD5-N-HA-1	*aod-5*, *pan-2*, A	This study. Transformation of PL23-40 with a hygromycin resistance plasmid carrying *aod-5* with triple HA tag coding sequence inserted after start codon
	Contains an ectopic copy of *aod-5* with N-terminal triple HA tag. Hygromycin resistant	
AOD5-C-Myc-4	*aod-5*, *pan-2*, A	This study. Transformation of PL23-40 with a hygromycin resistance plasmid carrying *aod-5* with triple myc tag coding sequence inserted before stop codon
	Contains an ectopic copy of *aod-5* with C-terminal triple myc tag. Hygromycin resistant	
DX13	*aod-2*, *aod-5*, *pan-2*	CNA33 × PL23-40
AOD2-C-Myc AOD5-N-HA	*aod-2*, *aod-5*, *pan-2*	This study. Cotransformation of DX13 with two hygromycin resistance plasmids. One containing *aod-2* with a triple myc tag inserted before the stop codon and one containing *aod-5* with a triple HA tag inserted after the start codon
	Contains an ectopic copy of *aod-5* with N-terminal triple HA tag and ectopic copy of *aod-2* with C-terminal triple myc tag	
96H9	Δ*aod-1*, A hygromycin resistant	FGSC # 18947
97B1	Δ*aod-2*, a hygromycin resistant	FGSC # 19465
1C3	Δ*aod-5*, a hygromycin resistant	FGSC # 11227

For localization studies and ChIP-seq analysis, strains were developed that expressed only tagged versions of either AOD2 or AOD5. The tags were either triple myc or triple hemagglutinin (HA). Tags were inserted at either the N- or C-terminus of the proteins (Table 1). Genes encoding the tagged proteins were individually inserted into a vector (Staben *et al.* 1989) carrying hygromycin resistance (HygR). The resulting plasmids were transformed into either an *aod-2* or *aod-5* mutant strain. Transformed strains were selected by plating on hygromycin-containing medium. Initial isolates were further purified by single colony isolation on hygromycin-containing medium. To demonstrate that a functional tagged AOD2 or AOD5 protein was present, the transformants were examined for their ability to grow at the wild-type rate in the presence of antimycin A (final concentration 0.5 µg/ml). Finally, they were examined for their ability to produce wild-type levels of AOX when grown in the presence of Cm. The isolates described in Table 1 were chosen for further work.

For coimmunoprecipitation experiments, a strain was developed that expressed tagged versions of both AOD2 and AOD5. The double *aod-2 aod-5* mutant strain DX-13 (Table 1) was cotransformed with a plasmid carrying an *aod-2* gene with a triple myc tag at its C-terminus and a plasmid with an *aod-5* gene with a triple HA tag at its N-terminus. Both plasmids also carried hygromycin resistance. Transformants were selected and analyzed as described above. The double-tagged strain chosen for analysis was AOD2-C-Myc AOD5-N-HA A1 (Table 1).

### Isolation of nuclei

The protocol for isolation of nuclei was as described previously (Talbot and Russell 1982), except that cells were broken while frozen in liquid nitrogen using a mortar and pestle, rather than a French press.

### Isolation of mitochondria

Mitochondria were isolated as previously described (Nargang and Rapaport 2007). When highly purified mitochondria were required for analysis in subcellular localization experiments, isolated mitochondria were further purified using sucrose flotation gradients (Lambowitz 1979).

### Cytosol and microsome isolation

To isolate cytosol and microsomes (postmitochondrial pellet; PMP) the mitochondrial isolation procedure (see *Isolation of mitochondria*) was followed. However, after pelleting the mitochondria by centrifugation, 1 ml of the supernatant was processed further. The supernatant sample was centrifuged at 48,000 rpm (27,915 × *g*) in a Beckman TLA-55 rotor for 1.5 hr at 4°. The pellet was suspended in 150 μl SEMP buffer [0.25 M sucrose, 10 mM MOPS (pH 7.2), 1 mM EDTA, 1 mM PMSF] as the microsomal or PMP fraction, whereas the supernatant was saved as the cytosolic fraction.

### Coimmunoprecipitation

To extract nuclear proteins, purified nuclei (100 μg protein) were suspended in 60 μl of suspension buffer [25 mM sucrose, 50 mM Tris-HCl (pH 7.5), 5 mM MgCl_2_, 10 mM CaCl_2_] and mixed with 60 μl of 0.4 M KCl containing 1 mM PMSF and protease inhibitors (final concentrations of 2 μg/ml aprotinin, 1 μg/ml leupeptin, and 1 μg/ml pepstatin A). The suspension was gently rocked for 2 hr at 4°. Insoluble material was removed by centrifugation at 13,000 rpm (16,060 × *g*) for 30 min at 4° in a Sorvall Biofuge fresco centrifuge. The supernatant containing salt-extracted proteins was loaded onto a desalting column (Zeba Spin Desalting column; Thermo Scientific, Rockford, IL) which was placed into a fresh 1.5 ml Eppendorf tube. The desalting column was centrifuged at 1500 × *g* (4000 rpm) for 2 min at 4° in a Sorvall Biofuge fresco centrifuge. The flow-through was immediately used in coimmunoprecipitation experiments with the Pierce ProFoundTM HA or c-Myc Tag IP/Co-IP kit (Thermo Scientific).

### ChIP-seq

ChIP-seq was performed on eight separate samples. Strain AOD2-C-HA-8 (expresses C-terminal triple HA–tagged AOD2) was grown in both the presence and absence of Cm. ChIP was done on both samples using an antibody against the HA tag. As controls, wild-type cells (NCN251) were also grown in both the presence and absence of Cm. ChIP was also done on these samples using the same antibody to the HA tag. Similarly, strain AOD5-C-Myc-4 (expresses C-terminal triple myc–tagged AOD5) was grown in the presence and absence of Cm and ChIP was performed on both samples using antibody against the myc tag. Again, control wild-type cultures were grown in the presence and absence of Cm and ChIP was performed using the same antibody against the myc tag. This resulted in four data sets, when controls were subtracted from experimental results.

The protocol for ChIP was as described (Guo *et al.* 2010). Cultures were inoculated with 2.5 × 10^8^ conidia in 100 ml Vogel’s medium, and were grown for 12 hr at 30° in the absence of Cm, or 14 hr in the presence of Cm. The final isolated ChIP-seq DNA was further treated using a Covaris S2 ultrasonicator to obtain DNA fragments with an average size of 200 bp for use in library construction. The sequence data for determination of binding sites of AOD2 following growth in the presence of Cm were obtained using an Illumina HiScan SQ with read lengths of 51 nucleotides. All other data were obtained using an Illumina HiSeq2000 with read lengths of 101 nucleotides. Covaris treatment, library construction, and DNA sequencing were performed by Delta Genomics (Alberta, Canada).

Reads were mapped to *N. crassa* Broad Institute Assembly 10 with BWA using default parameters (Li and Durbin 2009), and peaks were called using MACS2 (Model-based Analysis of ChIP-Seq) software (Zhang *et al.* 2008; https://github.com/taoliu/MACS/). The *q* value cutoff was set to 0.05. However, after selecting those peaks in each data set with fourfold or higher enrichment over the corresponding control, the highest *q* value in the data sets was 0.0022. The online program “Venny” was used to determine peaks in common to different data sets (Oliveros 2007–2015). Data were further analyzed by visual inspection of peaks in gbrowse (Stein *et al.* 2002; http://ascobase.cgrb.oregonstate.edu/cgi-bin/gb2/gbrowse/ncrassa_public/). Functional Catalogue (FunCat) analysis of genes (Ruepp *et al.* 2004) was done using FungiFun2 analysis (Priebe *et al.* 2015). Searches for consensus DNA binding sites were done using the SCOPE search tool (Chakravarty *et al.* 2007).

### RNA isolation and qPCR

Cultures were grown at 30° with constant shaking for 12 hr (in the absence of Cm) or 14 hr (in the presence of Cm). RNA was isolated using the QIAGEN (Ontario, Canada) RNeasy Plant Mini Kit. RNA integrity was measured using an Agilent 2100 Bioanalyzer system and the Agilent RNA 6000 Nano kit (Agilent Technologies). Only those samples that had an RNA integrity number value of >9 were used for cDNA synthesis. First-strand cDNA was synthesized using SuperScript III Reverse Transcriptase (Invitrogen). Reactions were performed using KAPA SYBR Fast qPCR reagents (KAPA Biosystems) and the StepOnePlus qPCR system (Applied Biosystems, Foster City, CA). Data were analyzed using StepOne software (Applied Biosystems). The β-tubulin gene was the internal control for ΔΔCt quantification. The qPCR data for each gene examined were obtained from four biological replicates, each with three technical replicates.

### General techniques

Electrophoresis of proteins (Laemmli 1970), Western blots (Good and Crosby 1989), and electroporation of conidia to transform *N. crassa* (Hoppins *et al.* 2007) were performed as described previously.

### Data availability

Strains and constructs are available upon request. Data are available from the SRA under BioProject accession no. PRJNA341311.

## Results

### Localization of AOD2 and AOD5

**Figure 1:**
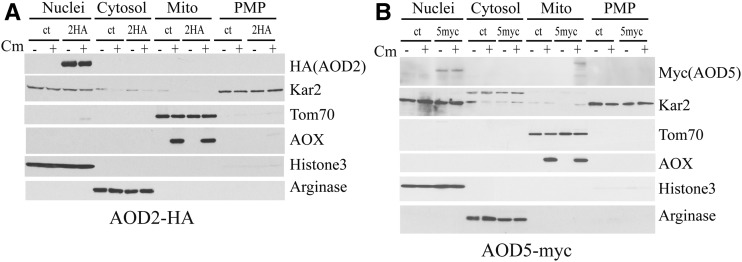
AOD2 and AOD5 are localized to the nucleus. (A) Strain AOD2-C-HA-8 (2HA) expresses an AOD2 protein with a triple HA tag at its carboxy-terminus. The strain was grown in both the presence and absence of Cm as was the control strain (ct) NCN251. Mycelium was harvested and different cellular fractions were isolated as described in *Materials and Methods*. Samples from the nuclear (nuclei, 20 µg protein), cytosolic (20 µg protein), mitochondrial (mito; 20 µg protein), and postmitochondrial pellet [(PMP) containing endoplasmic reticulum/microsomes, 20 µg protein] fractions were electrophoresed on SDS-PAGE gels along with molecular weight standards. Proteins were blotted to nitrocellulose membrane. The membrane was cut into strips according to the known molecular weights of the proteins to be detected as markers for the different fractions (nuclei: histone H3; cytosol: arginase; mitochondria: Tom70 and AOX; PMP: Kar2) or the HA-tagged AOD2 protein. Antibodies used for detection are shown on the right side of the blots. (B) As in (A), except that strain AOD5-C-Myc-4 (5myc), which expresses AOD5 containing a triple myc tag at its carboxy-terminus, was used.

Although *N. crassa* contains two genes encoding AOX, *aod-1* and *aod-3*, no conditions have been identified that result in the expression of *aod-3* (Tanton *et al.* 2003). Thus, any measurable AOX activity in the organism is derived from the AOD1 protein. The *aod-1* gene is transcribed at low levels under normal growth conditions. However, growth in the presence of inducers increases expression of both the transcript and the protein many fold (Tanton *et al.* 2003; Chae *et al.* 2007b). Increased expression is dependent on the transcription factors, AOD2 and AOD5. EMSA analysis demonstrated that the two proteins bound as a heterodimer to an AIM upstream of the *aod-1* gene *in vitro* (Chae *et al.* 2007b). To further understand the mechanism by which these transcription factors induce *aod-1* expression, we wished to determine if the amount or subcellular location of either AOD2 or AOD5 was affected by growth of cells in AOX-inducing conditions compared with noninducing conditions. To monitor the location of the proteins, we developed strains expressing AOD2 or AOD5 proteins with triple HA or myc tags at either their N- or C-terminus. The tagged strains were grown in the presence (AOX-inducing conditions) or absence (AOX-noninducing conditions) of Cm. Cells were harvested and various subcellular fractions were isolated. Analysis of strains expressing a C-terminal HA-tagged AOD2 (AOD2-HA) and a C-terminal myc-tagged AOD5 (AOD5-myc) revealed that both proteins were only detectable in the nuclear fraction and their localization was not influenced by growth in the presence or absence of Cm (Figure 1, A and B). The levels of the proteins did not appear to change substantially between the two growth conditions. To examine the possibility that the tagged proteins were simply mislocalized due to the location of the tags on the C-terminus, we also examined subcellular fractions from a strain expressing an N-terminal HA-tagged AOD2 (HA-AOD2) and a strain expressing an N-terminal HA-tagged AOD5 (HA-AOD5). Localization results were virtually identical to those observed for the C-terminal tagged proteins (Supplemental Material, Figure S1).

**Figure 2:**
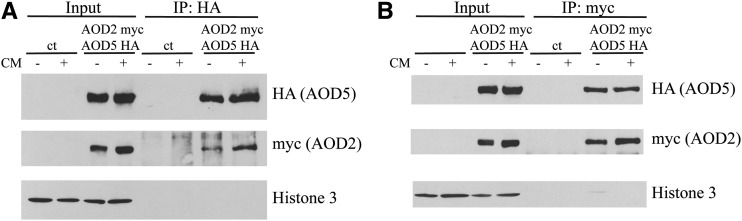
AOD2 and AOD5 associate with each other *in vivo*. Control strain NCN251 (ct) and strain AOD2-C-Myc AOD5-N-HA, which expresses both an AOD2 protein carrying a triple myc tag at its carboxy-terminus and an AOD5 protein carrying a triple HA tag at its amino-terminus, were grown both in the presence and absence of Cm. Mycelium was harvested and nuclear proteins were isolated (Input). Immunoprecipitation (IP) was performed with either anti-HA (A) or anti-myc antibodies (B) bound to agarose beads. The beads were washed and bound proteins were eluted as described in *Materials and* *Methods*. The elution fractions were subjected to SDS-PAGE and separated proteins were blotted to nitrocellulose membrane. The membranes were probed with the antibodies shown to the right of the panels. Histone 3 was used as a control for loading and purification.

Our previous *in vitro* findings have shown that protein fragments containing the DNA-binding domains of AOD2 and AOD5 act as a heterodimer when binding DNA. To demonstrate that the two full-length proteins associated *in vivo*, we performed pull-down experiments using proteins extracted from nuclei isolated from a strain expressing differentially tagged versions of both proteins (AOD2-myc and HA-AOD5). We found that either tagged protein would coimmunoprecipitate the other (Figure 2), demonstrating that the two full-length proteins do associate *in vivo*. Furthermore, the results were virtually identical regardless of whether cultures were grown in the presence or absence of Cm (Figure 2). Taken together, these data suggest that the expression of *aod-1* is mediated by an AOD2/AOD5 heterodimer. Changes in growth conditions do not result in changes of the levels or localization of AOD2 and AOD5. Furthermore, at least a fraction of each protein is associated in a heterodimer under both inducing and noninducing conditions.

### AOD2 and AOD5 are required for optimal growth in different carbon sources

It has been shown in other filamentous fungi, *A. nidulans* (Suzuki *et al.* 2012) and *P. anserina* (Sellem *et al.* 2009), that the orthologs of AOD2 and AOD5 are not only required for the expression of AOX, but also for genes involved in gluconeogenesis. To evaluate this in *N. crassa*, we measured transcript levels of genes involved in gluconeogenesis in strains lacking either AOD2 or AOD5 following growth in different carbon sources. Since defects in ability to utilize certain carbon sources might affect the growth of the mutants, we also followed the growth rate of the mutant stains under these conditions. We had previously been unable to detect differences in colonial growth rate between controls and mutant *aod-2* or *aod-5* cells when tested on solid Vogel’s medium containing sorbose (Descheneau *et al.* 2005). Therefore, in the current study, we measured growth in liquid medium containing the different carbon sources. In addition, since the classic retrograde regulation mutants of *Saccharomyces cerevisiae* have defects in nitrogen metabolism (Liu and Butow 2006), we used synthetic crossing medium as the source of inorganic salts since it contains a reduced overall nitrogen concentration relative to the standard Vogel’s salts and totally lacks ammonium (see *Materials and* *Methods*), which is a preferred nitrogen source for *Neurospora* (Marzluf 1997).

**Figure 3:**
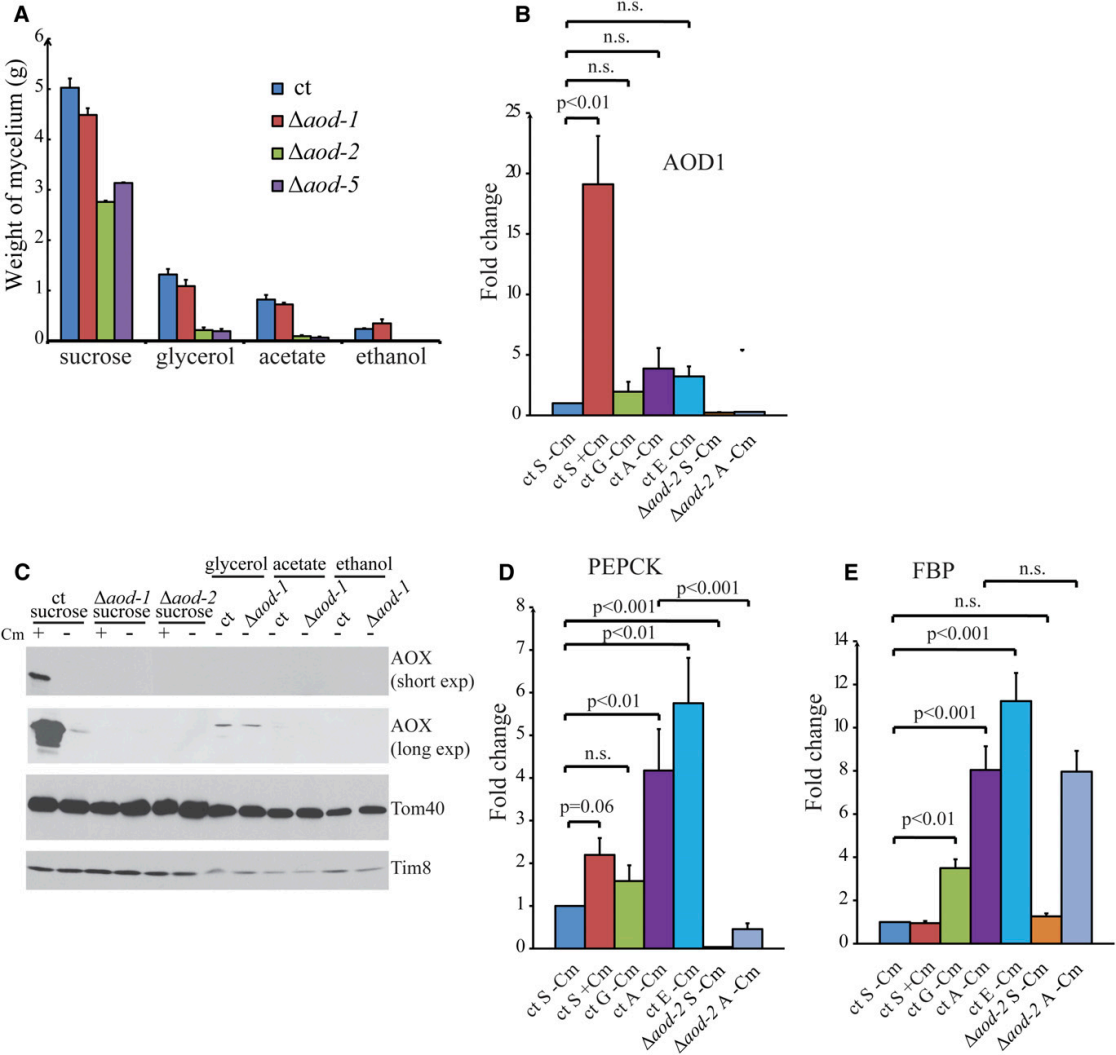
Effect of carbon source on growth and transcript levels. (A) The control strain (ct) NCN251 and the Δ*aod-1*, Δ*aod-2*, and Δ*aod-5* knockout strains were grown in low nitrogen-containing medium with either 44 mM sucrose, 217 mM glycerol, 150 mM sodium acetate, or 217 mM ethanol as the carbon source. For the different carbon sources, mycelium was harvested and weighed from these cultures after 20, 72, 72, and 96 hr of growth, respectively. The values shown are the average of three cultures of each strain and carbon source. Error bars show the SEM. (B) The ct NCN251 and the Δ*aod-2* knockout strain were grown on different carbon sources, as described in (A) (S, sucrose; G, glycerol; A, acetate; E, ethanol), with or without Cm present in the medium as indicated. Mycelium was harvested and total RNA prepared. RT-qPCR was performed for AOD1 mRNA using cDNA generated from the RNA. Error bars shows the SEM. The Student’s *t*-test was used to compare data between two different conditions. *p* ≥ 0.05; n.s., no significant difference. (C) Mitochondria were isolated from the ct NCN251 and the knockout strains Δ*aod-1* and Δ*aod-2* grown in the presence or absence of Cm, and in different carbon sources as indicated at the top of the panel. Mitochondrial proteins were subjected to SDS-PAGE and the proteins were transferred to nitrocellulose membrane. The blots were probed with the antibodies indicated on the right. Two different exposure times are shown for AOX (short exposure, long exposure). Tom40 and Tim8 serve as mitochondrial marker loading controls. (D and E) As for (B), except that the RT-qPCR was performed for PEPCK and FBP mRNA, respectively.

When sucrose was the carbon source, the mutant strains grew at ∼50–60% of the rate of the control strain (Figure 3A). However, when either acetate or glycerol was used as the carbon source, the growth rate of the mutant strains was more severely affected, decreasing to 10–20% of the control on glycerol or acetate and to virtually no growth when ethanol was used as the carbon source. These differences were not due to loss of the ability to produce the AOX AOD1 protein, since a knockout of AOD1 grew at near wild-type rates on all media (Figure 3A).

To determine if the reduced growth on poor carbon sources in the mutant strains was due to an effect on the expression of genes required for gluconeogenesis, we performed qPCR analysis on RNA isolated from an *aod-2* knockout strain and a control following growth under various conditions. Levels of the transcripts for AOD1, as well as the gluconeogenic enzymes PEPCK and FBP, were determined. As expected, the greatest increase in the *aod-1* transcript was seen in the cultures grown in the presence of Cm (Figure 3B). Slight increases in *aod-1* mRNA were also seen following growth in poor carbon sources compared with the amounts present in uninduced sucrose cultures, but these were not found to be statistically significant. Furthermore, no AOD1 protein was seen in mitochondria from cultures grown in poor carbon sources (Figure 3C). For PEPCK mRNA (Figure 3D), a small increase due to growth in the presence of Cm was observed, but statistical significance was borderline (*p* = 0.06). Larger increases were seen following growth in acetate and ethanol. Growth in glycerol as the carbon source had virtually no effect on the level of PEPCK transcript, probably because glycerol enters the gluconeogenic pathway after the action of PEPCK. The mRNA for FBP (Figure 3E) showed no increase in medium containing Cm, but was significantly increased in all three of the poor carbon source cultures compared with the sucrose culture. Interestingly, both AOD1 and PEPCK transcripts were severely reduced in cultures lacking AOD2, but FBP transcript abundance was unaffected by the absence of AOD2. We conclude that expression of the gene encoding PEPCK in *N. crassa* requires AOD2, and by extension AOD5, but FBP expression does not.

### ChIP-seq analysis of AOD2 and AOD5 binding

The reduced growth of the *aod-2* and *aod-5* knockouts in sucrose medium without Cm suggested a wider role of the two transcription factors than simply gluconeogenesis and *aod-1* induction. Furthermore, our results suggested that AOD2 and AOD5 were not required for transcription of the gene encoding FBP, in contrast to results in *A. nidulans* (Suzuki *et al.* 2012) and *P. anserina* (Sellem *et al.* 2009). For these reasons, we wished to gain deeper insight into the function and roles of AOD2 and AOD5. We used a ChIP-seq approach to identify binding sites of the two proteins in the *Neurospora* genome. Four separate experiments were performed to examine the binding of AOD2 and AOD5 in cultures grown in either the presence or absence of Cm. Since antibodies against these proteins were not available, we utilized commercially available antibodies against the HA and myc antigens. This enabled immunoprecipitation from the strains expressing the AOD2-HA– and AOD5-myc–tagged proteins used in the localization experiments described above. To help eliminate false positive results, ChIP was also done using the same antibodies, on a control strain that did not contain tagged proteins. The ChIP-seq experiments and the number of sequence reads from each are shown in Table 2. The initial data from each experiment following MACS2 (Zhang *et al.* 2008) analysis are shown in File S1, File S2, File S3, and File S4. Read alignments to the *N. crassa* genome can be viewed at http://ascobase.cgrb.oregonstate.edu/cgi-bin/gb2/gbrowse/ncrassa_public/. Further investigation was done on all peaks with fourfold or greater enrichment relative to the appropriate control matched for growth condition and antibody.

**Table 2:** ChIP experiments

ChIP Experiment	AOD2 Protein	AOD5 Protein	Growth Condition	Antibody Used	Mapped Reads
FN1	AOD2-HA	Wild type	+Cm	Anti-HA	9,864,194
FN2	Wild type	Wild type	+Cm	Anti-HA	6,920,439
FN3	Wild type	AOD5-Myc	+Cm	Anti-Myc	3,341,525
FN4	AOD2-HA	Wild type	−Cm	Anti-HA	1,438,498
FN5	Wild type	Wild type	−Cm	Anti-HA	887,801
FN6	Wild type	AOD5-Myc	−Cm	Anti-Myc	2,016,155
FN7	Wild type	Wild type	−Cm	Anti-Myc	1,281,599
FN8	Wild type	Wild type	+Cm	Anti-Myc	2,511,851

**Figure 4:**
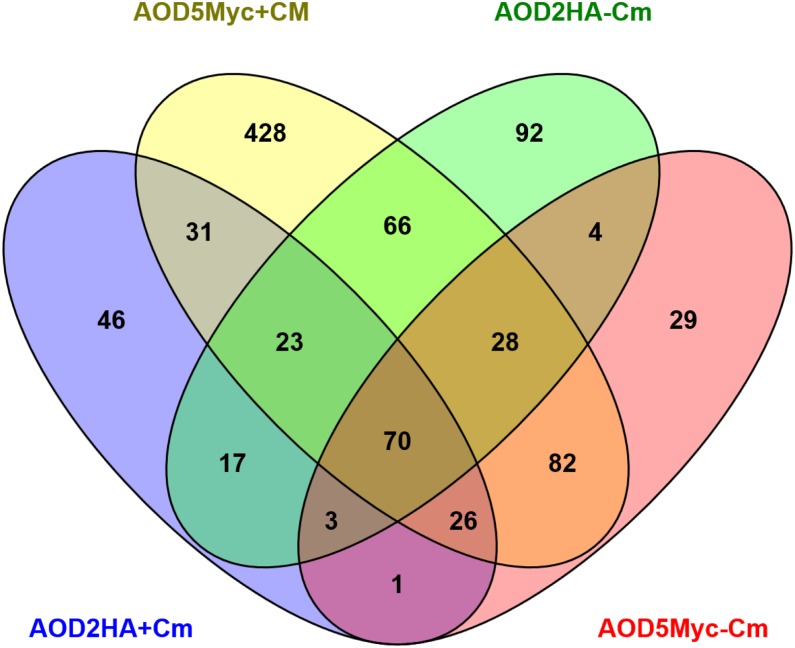
Genes in common to all four experimental conditions examined by ChIP-seq analysis. Genes in each ChIP that were associated with binding greater than fourfold, compared with the nontagged wild-type control strain in each experimental condition, were identified. These were compared using the online Venn diagram generating program, Venny (Oliveros 2007–2015). The individual ChIP experiments are indicated beside the appropriate oval. For further analysis, Venny also lists the genes occurring in any segment of the Venn diagram.

Given that AOD2 and AOD5 have been shown to act as a heterodimer and that both proteins were found in the nuclear fraction, regardless of whether cultures were grown in inducing or noninducing conditions, we concentrated on peaks in common to all four experiments as the most likely to be authentic AOD2/AOD5 binding sites. Comparison of the data sets from all four experiments, using the Venn diagram list comparison program Venny (Oliveros 2007–2015), revealed 70 peaks in common to all four conditions (Figure 4). Five of the peak sets were found to occur between the 3ʹ ends of two genes. It is conceivable that antisense transcripts arise from these genes in an AOD2/AOD5-dependent fashion, since *N. crassa* does produce antisense transcripts (Arthanari *et al.* 2014) and at least one of these is known to have a regulatory function (Kramer *et al.* 2003). However, we chose to focus our investigation on genes with binding sites upstream of their coding regions. Thus, these five sets were not considered further. For the remaining 65 peaks (Table 3) found in presumptive gene promoters, 32 were associated with one gene in the proper orientation, while in 33 cases, the genes were oriented such that the binding sites were upstream of divergently transcribed genes. For these 33, we cannot assign transcriptional control by AOD2/AOD5 to one of the genes without further investigation.

**Table 3:** Peaks in common to all four ChIP-seq experiments with over fourfold enrichment relative to controls

Gene(s) in Correct Orientation Relative to ChIP Peaks	qPCR Result*^a^*	AIM Sequence*^b^*	AIM Summit*^c^*
NCU00576 (hypo)	Nd	Y	N
NCU00577 (hypo)	Nd	Y	N
NCU00682 (Ser-Thr protein kinase)	Nd	Y	Y
NCU00865 (oxalate decarboxylase)	Nd	Y	Y
NCU00866 (DUF 1275)	Nd	Y	Y
NCU01195 (gdh)	No effect	Y	Y
NCU01224 (26S protease reg subunit)	No effect	N	Na
NCU01225 (ubiquitin conj enzyme)	No effect	N	Na
NCU01744 (glutamate synthase)	Nd	Y	Y
NCU01742 (hypo)	Nd	Y	Y
NCU01808 (cytochrome *c*)	2+, 5+	Y	Y
NCU01807 (hypo)	Nd	Y	Y
NCU01834 (hypo)	Nd	Y	Y
NCU01944 (hypo)	Nd	N	Na
NCU02092 (hypo)	Nd	Y	Y
NCU02474 (Tom5)	No effect	Y	Y
NCU02475 (glycine dehydrogenase)	2+, 5+	Y	Y
NCU02496 (M phase inducer phosphatase 3)	Nd	Y	Y
NCU02497 (hypo)	Nd	Y	Y
NCU02514 (ATPase-1)	No effect	N*^d^*	Na
NCU02549 (processing enhancing protease)	Nd	Y	Y
NCU02550 (α-galactosidase)	Nd	Y	Y
NCU02720 (hypo)	Nd	Y	Y
NCU02732 (hypo)	No effect	N	Na
NCU03118 (hypo)	Nd	Y	Y
NCU03120 (hypo)	Nd	Y	Y
NCU03257 (ammonium transporter MEP1)	Nd	Y	Y
NCU03364 (DENN domain)	Nd	Y	Y
NCU03365 (hypo)	Nd	Y	Y
NCU03408 (hypo)	2+, 5+	Y	Y*^e^*
NCU03409 (RNA lariat debranching)	No effect	Y	Y
NCU03466 (hypo)	Nd	Y	Y
NCU03548 (hypo)	Nd	Y	Y
NCU03549 (hypo)	Nd	Y	Y
NCU03593 (kaleidoscope-1)	Nd	Y	Y
NCU03651 (NADP-dependent malic enzyme)	2+, 5+	Y	Y
NCU03749 (hydroxyacyl glutathione hydrolase)	2−	N	Na
NCU04307 (MSF1)	No effect	Y	Y
NCU04569 (5-oxoprolinase)	2+, 5+	Y	Y
NCU04730 (post-transcript silencing protein QDE-2)	No effect	Y	N
NCU04801 (hypo)	Nd	Y	N
NCU04874 (aod-3)	5−	Y	Y
NCU04953 (penicillopepsin)	Nd	Y	N
NCU04954 (hypo)	Nd	Y	N
NCU04986 (hypo)	Nd	N	Na
NCU11067 (hypo)	Nd	N	Na
NCU05202 (mt K+ H+ exchange)	No effect	Y	N
NCU05203 (hypo)	No effect	Y	N
NCU05299 (NADH-ubiquinone reductase 29.9)	No effect	N	Na
NCU05390 (mt phosphate carrier)	Nd	Y	Y
NCU05391 (hypo)	Nd	Y	Y
NCU05477 (hypo)	Nd	Y	Y
NCU11913 (hypo)	Nd	Y	Y
NCU05616 (arsenite S-adenosyl methyl transferase)	Nd	N*^f^*	Na
NCU05989 (hypo)	Nd	Y	Y
NCU06083 (hypo)	Nd	Y	N
NCU06230 (Ser/Thr kinase)	2−, 5−	Y	Y
NCU06381 (cell wall endoglucanase)	Nd	Y	Y
NCU06382 (ABC transporter)	2+, 5+	Y	Y
NCU06387 (hypo)	Nd	Y	Y
NCU06389 (DUF 625 domain)	Nd	Y	Y
NCU06424 (amino methyl transferase)	Nd	Y	Y
NCU06425 (hypo)	Nd	Y	Y
NCU06426 (hypo)	Nd	Y	Y
NCU06724 (glutamine synthase)	Nd	Y	Y
NCU06939 (endosome assoc ubiquitin isopeptidase)	No effect	Y	Y
NCU06940 (hypo)	2+, 5+	Y	Y
NCU07098 (hypo)	Nd	Y	N
NCU07099 (hypo)	Nd	Y	N
NCU07166 (hypo)	Nd	Y	Y
NCU07167 (isoflavone reductase)	Nd	Y	Y
NCU07327 (hypo)	Nd	Y	Y
NCU07530 (transporter Smf2)	Nd	Y	N
NCU07531 (Cu transporting ATPase)	Nd	Y	N
NCU07668 (MFS multidrug transporter)	Nd	Y	N
NCU11717 (hypo)	Nd	Y	N
NCU07676 (hypo)	Nd	N	Na
NCU07678 (hypo)	No effect	N	Na
NCU07807 (fructose bisphophatase aldolase)	No effect	Y	Y
NCU07941 (aspartate amino transferase)	Nd	Y	Y
NCU07942 (hypo)	Nd	Y	Y
NCU07953 (aod-1)	2+, 5+	Y	Y
NCU07954 (hypo)	Nd	Y	Y
NCU08674 (pentatricopeptide repeat protein)	2+, 5+	Y	Y
NCU08877 (glycine cleavage system H)	No effect	Y	Y
NCU08878 (Tem1)	No effect	Y	Y
NCU08940 (complex III protein)	Nd	Y	Y
NCU08941 (Ca binding mt carrier)	Nd	Y	Y
NCU08946 (hypo)	No effect	N	Na
NCU08947 (complex III protein)	2+, 5+	N	Na
NCU08976 (fatty acid elongase)	Nd	Y	Y
NCU08977 (long chain fatty alcohol oxidase)	Nd	Y	Y
NCU09656 (carboxymethyl butenelidase)	Nd	Y	Y
NCU11414 (plasma membrane Zn transporter)	No effect	Y	Y
NCU09873 (PEPCK)	2+, 5+	Y	Y
NCU10007 (malate synthase)	2+, 5+	Y	Y
NCU10894 (hypo)	Nd	Y	Y
NCU12093 (phospholipase D)	Nd	Y	Y

Alternating gray and white rows indicate one or two genes in the correct orientation to a set of peaks found in all four experiments. NCU numbers are followed by known gene functions where applicable. General information about *N. crassa* genes is available at FungiDB (http://fungidb.org/fungidb/). hypo, hypothetical protein

a Nd, not determined. Numbers indicate either AOD2 or AOD5. The plus sign indicates that protein is a positive regulator of the gene. The minus sign indicates that protein is a negative regulator of the gene.

b Y (yes), one or more AIM sequences present in upstream intergenic region of gene(s). N (no), no AIM sequence present in upstream intergenic region.

c Alignment of at least one AIM sequence with at least one ChIP-seq summit. Y (yes), AIM sequence at or near ChIP-seq summit. N (no), AIM sequence not found at or near summit. Na, not applicable.

d No AIM sequence in intergenic region but AIM present in nearby coding region and does align with ChIP-seq peak summit.

e Summit and AIM match, but only for the AOD5 peaks. Summits of the AOD2 peaks match the repeat sequence consensus (see AOD2 binds to a repeat sequence in the genome).

f As footnote *c*, but aligns only with shoulders of summits.

**Figure 5:**
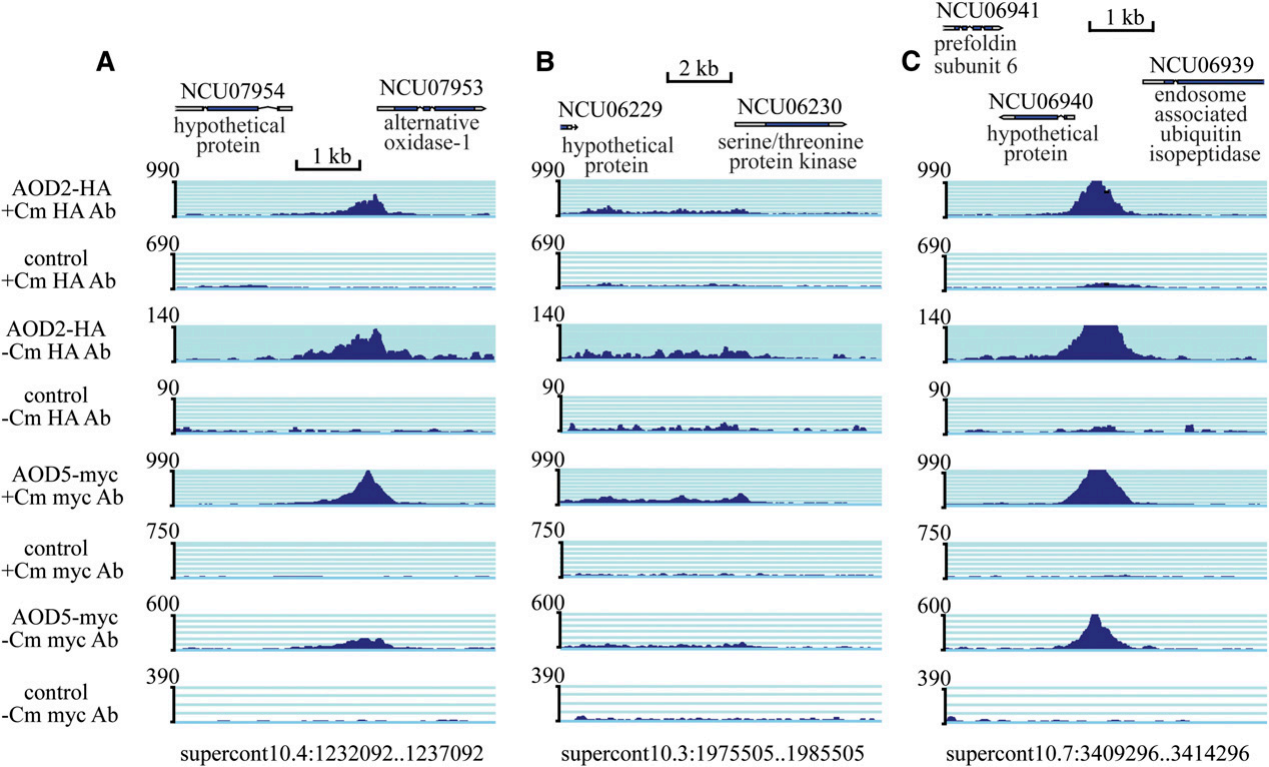
Variation in peaks called by MACS2. Snapshots from gbrowse, where peaks from ChIP-seq experiments have been aligned with the *N. crassa* genome. The ratios of the *y*-axis in different immunoprecipitations were assigned based on the number of mappable sequence reads obtained in the individual experiments for the HA or myc antibodies. However, no attempt to standardize between the different antibodies was made since their relative efficiencies for immunoprecipitation may differ. (A) Peaks found in the upstream region of the *aod-1* gene (encoding AOX, NCU07953). The position of genes in the region is shown at the top. The individual experiments are listed on the left with respect to the tagged protein in the strain used, the growth condition (±Cm), and the antibody (Ab) used for immunoprecipitation. The control ChIP-seq experiments were performed on strain NCN251 (expresses no HA- or myc-tagged proteins), exactly as with the strains expressing tagged proteins. (B) As in (A), except the region containing the NCU06229 and NCU06230 genes is shown. (C) As in (A), except the region containing the NCU06939 and NCU06940 genes is shown. In (B) and (C), the *y*-axis scale was not adjusted so that the difference in height of the peaks relative to those in (A) would be emphasized.

Visual inspection of the 65 AOD2/AOD5 binding sites revealed a broad range of quality and characteristics in the peaks. For example, many peaks appeared robust and spanned roughly 0.5–1 kb at the binding site in all four experiments, as for the AOX-encoding *aod-1* gene (NCU07953) (Figure 5A). The peaks at several other sites were relatively smaller and/or were broad, sometimes extending over several kb, as for NCU06230, which encodes a Ser/Thr protein kinase (Figure 5B). The most robust peaks in all four experiments, as judged by visual inspection, were seen in the region between the endosome-associated ubiquitin isopeptidase gene (NCU06939) and the hypothetical protein-encoding gene, NCU06940 (Figure 5C). Though there is variation among the different sets of peaks with respect to amount of binding, in virtually all cases, binding of both proteins was observed following growth in both the presence or absence of Cm.

**Figure 6:**
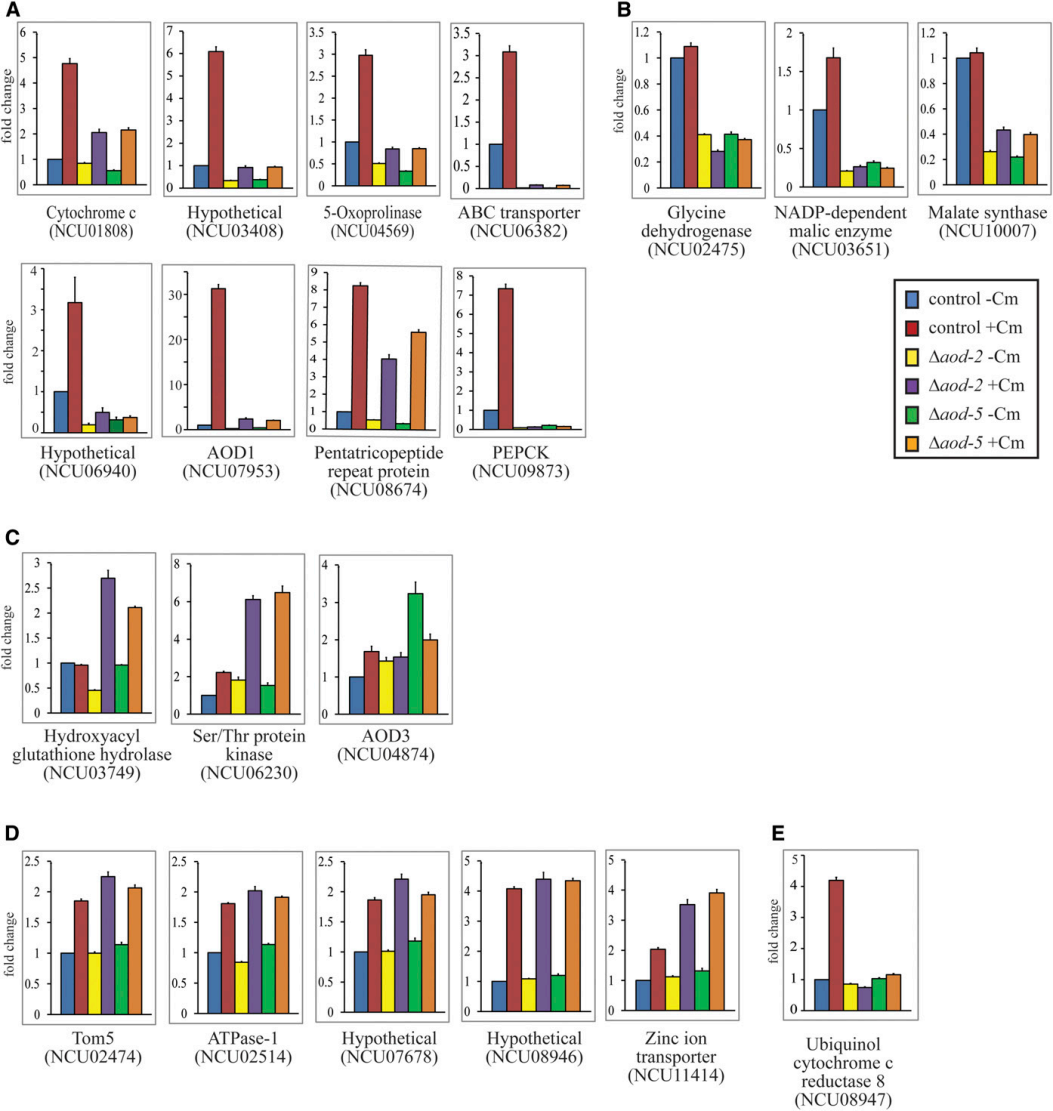
Genes tested by qPCR from among the common set of 65. The control strain (ct) NCN251 and the Δ*aod-2* and Δ*aod-5* knockout strains were grown in both the presence and absence of Cm. Total RNA was isolated from each of the strains, and mRNA was converted to cDNA. qPCR was performed as described in *Materials and* *Methods*. The data for the strains and conditions used are indicated by different colors, as shown on the figure. The results shown are the average of four biological replicates for each strain, each of which was determined using three technical replicates. In each case, the value for the control grown without Cm was set as one and the other data are represented as the fold change relative to that standard. Error bars show the SEM. (A) Genes found to require AOD2 and AOD5 for maximal expression and also show induction by Cm. (B) As in (A), but expression of these genes is not induced twofold or greater by Cm. (C) Genes found to be negatively regulated by AOD2 and/or AOD5. (D) Genes exhibiting only a Cm effect on transcription that does not depend on AOD2 or AOD5. (E) Gene exhibiting induction by Cm that does require AOD2 and AOD5. Expression of this gene in the absence of Cm does not require AOD2 or AOD5.

For 33 of the 98 genes listed in Table 3, we used a qPCR approach to determine if their expression was dependent on AOD2 and/or AOD5. RNA was isolated from wild-type, Δ*aod-2*, and Δ*aod-5* cells, each grown in the presence and absence of Cm. Eleven of the genes examined showed greater than twofold decreased expression in cells lacking either AOD2 or AOD5 in both of the growth conditions (Figure 6), suggesting a positive regulatory role for the transcription factors. Eight of these 11 genes also showed greater than twofold increase in expression in the presence of Cm in wild-type cells (Figure 6A), while three showed little to no change in expression due to the presence of Cm (Figure 6B). Thus, all 11 genes require AOD2 and AOD5 for maximal expression both in the presence and absence of Cm, but for a subset of three, their expression is virtually unaffected by the presence of Cm. Two genes showed a twofold or greater increase in expression in the absence of either AOD2 or AOD5 when cells were grown in the presence of Cm (Figure 6C). For these genes, the transcription factors appear to play a negative role in expression. One gene (NCU04874) showed increased expression in the absence of Cm in cells lacking AOD5 (Figure 6C). Interestingly, NCU04874 represents the *aod-3* gene. We had previously shown that *aod-3* encodes a second, potentially functional AOX, but could find no evidence of its expression on Northern blots under any condition tested (Tanton *et al.* 2003). Our data show that both AOD2 and AOD5 bind at the promoter region of *aod-3*, and suggest that at least AOD5 may play a role in repressing transcription. Thus, it appears that AOD2 and AOD5, most likely acting as a heterodimer, can serve as either positive or negative regulatory factors.

Examination by qPCR of the expression of 13 genes from Table 3 revealed no obvious effect with regard to the presence or absence of AOD2 or AOD5, nor was an effect seen following growth in the presence or absence of Cm (Figure S2). Conceivably, unknown conditions may elicit a response in these genes which is mediated by AOD2 and AOD5, with or without contributions from other protein factors.

Unexpectedly, five genes showed less than twofold change in expression in the absence of AOD2 or AOD5, but did show two to fourfold increases in expression when cells were grown in the presence of Cm, regardless of whether AOD2 or AOD5 was present (Figure 6D). Two of the genes in this category, NCU02474 [encoding TOM5, a component of the mitochondrial TOM complex (Schmitt *et al.* 2005; Sherman *et al.* 2005)] and NCU08946 (hypothetical protein), were adjacent to divergently transcribed genes (NCU02475 and NCU08947, respectively) whose expression was reduced by loss of AOD2 and AOD5 (Figure 6, B and E). Whether there is a relationship that affects the expression of these genes that are arranged in divergent orientation is unknown. NCU08947 is unusual because the ChIP-seq peaks appear to be within an intron near the 5′ end of the gene and they were not associated with an AIM. The expression of NCU08947 was not affected by loss of AOD2 or AOD5 in the absence of Cm, but both proteins were required for maximal expression in the presence of Cm (Figure 6E).

**Figure 7:**
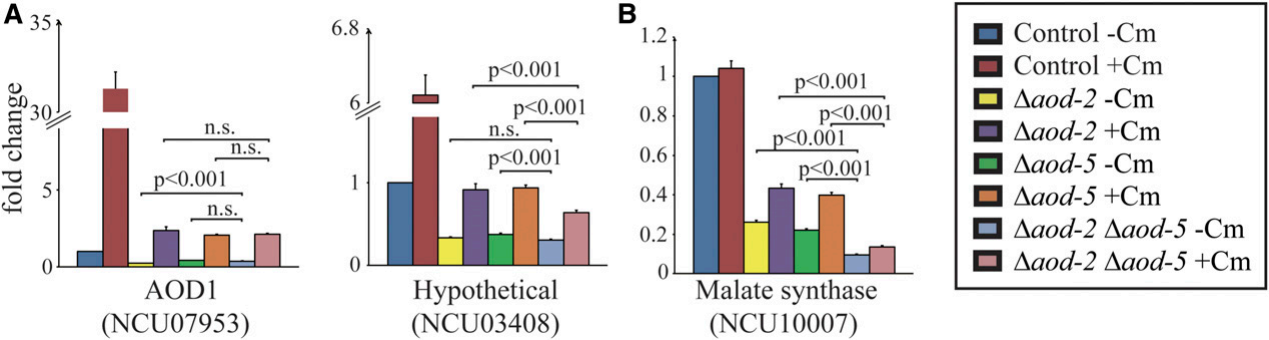
Comparison of transcript levels in a double *aod-2 aod-5* mutant strain *vs.* single mutant strains. The results for the transcripts from the indicated genes, as shown in Figure 6, were compared with the results from the double mutant, as described in the legend to Figure 6. The Student’s *t*-test was used to compare data between two different conditions. (A) Two genes with less than twofold difference between single and double mutants. (B) One gene showing a greater than twofold decrease in expression in the double mutant as compared with the single mutants. *p* ≥ 0.05; n.s., no significant difference.

For most of the genes in Figure 6, A and B, we observed a slight induction of transcription in the presence of Cm, even in the absence of either AOD2 or AOD5. It seemed possible that this might be due to the action of the other remaining transcription factor in the single mutant strains. To test this, we created a Δ*aod-2* Δ*aod-5* double mutant strain and examined transcript levels for certain genes requiring AOD2 and AOD5 for expression. There was virtually no change in transcript levels when the single mutants were compared with the double mutants (Figure 7A) for NCU07953 (AOD1) and NCU03408 (hypothetical protein). Although some small differences were found to be statistically significant, the overall changes were often much less than twofold and the biological significance seems questionable. However, the gene NCU10007 (malate synthase) appeared to have a slight reduction of transcription in the double mutant strain (Figure 7B). Overall, the slight induction in the presence of Cm still remained in the double mutant strain, arguing for the involvement of other factors in the response to the induction signal(s) produced by growth in the presence of Cm.

Previous EMSA data has shown that the AIM (CGGN_7_CGG), found 170 bp upstream of the predicted *aod-1* start codon, bound AOD2 and AOD5 only when both proteins were present, suggesting that they bind as a heterodimer. The element was also required for maximal expression of the *aod-1* gene in *N. crassa* (Chae *et al.* 2007a,b). Thus, we examined the intergenic upstream regions of the 65 peak sets (98 genes) in Table 3 for the presence of this sequence, using the SCOPE search tool (Chakravarty *et al.* 2007). One or more AIM sequences were found (Table 3) for 55 peak sets (84 genes). We also determined the position of the AIM sequence relative to the summit of the 55 ChIP-seq peak sets. Although this assessment is difficult since many peak sets have multiple summits and more than one AIM, we found that there was good concordance of at least one AIM with one summit in the intergenic regions in 46 peak sets (69 genes) (Figure S3 and Table 3). All of the genes for which AOD2 and AOD5 play a positive regulatory role, as shown in Figure 6, A and B, were found to have an AIM sequence associated with at least one peak summit, with the exception of NCU03408. The latter gene is a special case since it is also associated with another consensus sequence found in a repeated sequence that appears in several places in the genome (see AOD2 binds to a repeat sequence in the genome). For peak sets containing no AIM, or where an AIM exists but does not coincide with a peak, binding of AOD2 and AOD5 generally occurs at a low level, with the exception of some sites that appear to bind AOD2 more efficiently than AOD5 (see AOD2 binds to a repeat sequence in the genome).

**Figure 8:**
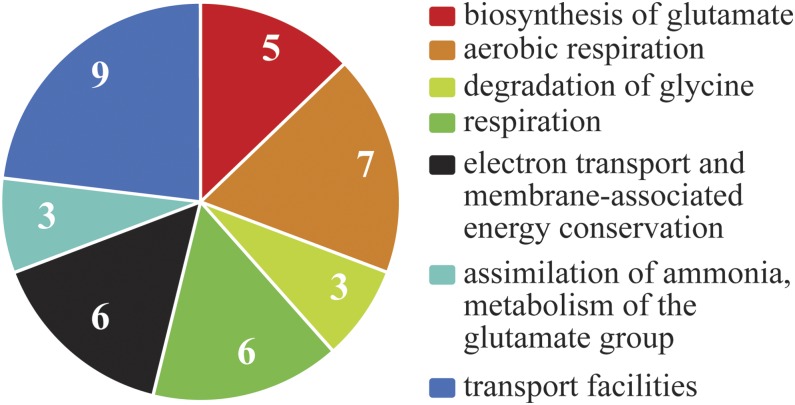
Over-represented genes in functional categories. Genes in the group of 65 peak sets common to all four ChIP-seq experiments (Table 3) that have known functions or domains were compared with genes of known function in the *N. crassa* genome, using FungiFun2 (Priebe *et al.* 2015). The seven different over-represented functional categories are indicated.

The 98 genes in Table 3 include 42 hypothetical proteins and two proteins with domains of unknown function. The remaining 54 genes that are associated with a known function or domain were examined for related functions using the FunCat classification (Ruepp *et al.* 2004) in FungiFun2 (Priebe *et al.* 2015) (https://elbe.hki-jena.de/fungifun/fungifun.php). Of those 54, 23 were found to be in seven classes that were over-represented in the sample, relative to known genes in the *N. crassa* genome, with 11 of the 23 genes listed in two or more functional categories (Figure 8 and Table S1). The seven classes fall into three of the main FunCat categories: energy, metabolism, and cellular transport. Eleven of the genes found in the FunCat analysis were among those analyzed by qPCR for expression differences in the absence of AOD2 or AOD5. Seven were found to have altered expression while no effects were observed for four others. However, the appearance of these genes in functionally enriched categories argues that the binding of the transcription factors may be significant, but that proper conditions for altering expression have not yet been discovered.

**Figure 9:**
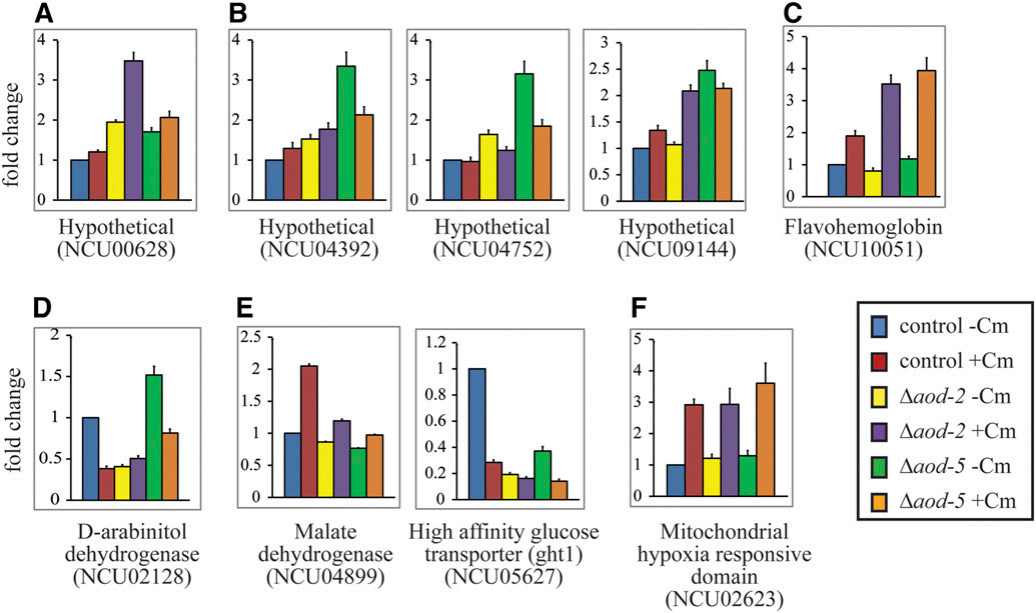
Other genes affected by AOD2 and/or AOD5. Genes from categories of the Venn diagram that were not in the group common to all four ChIP-seq experiments were examined for expression by qPCR, as described in the legend to Figure 6. (A) Increased expression in the absence of AOD2 when grown in the presence of Cm. (B) Increased expression in the absence of AOD5 without CM. (C) Increased expression without AOD2 or AOD5 in the absence of Cm. (D) Decreased expression without AOD2. (E) Decreased expression without AOD2 or AOD5 in the presence of Cm (NCU04899) or decreased expression in the presence of Cm and dependence on AOD2 and AOD5 for expression in the absence of Cm (NCU05627). (F) Cm increases expression but no effect with loss of AOD2 or AOD5.

Inspection of peaks from other regions of the Venn diagram (Figure 4) where three of the four experiments had peaks in common, where peaks were specific for AOD-2 or AOD-5, or where peaks were specific for the presence or absence of Cm revealed 12 other sets of peaks that appeared robust and/or were in proximity to genes with a relevant metabolic function (Table 4). The expression of the 14 genes that are in the correct orientation relative to these peaks was examined by qPCR to determine the effects of loss of AOD2 or AOD5, and the presence of Cm on expression. We also included the NCU10051 gene (flavohemoglobin) in this study as an example of a gene identified in the *P. anserina* microarray study that was not found in any of the groupings described here using the criteria chosen. Six genes showed no differences greater than twofold in any pairwise comparisons (Figure S4). One of the genes in this category was NCU04797, which encodes *N. crassa* FBP. Nine of the genes examined showed an over twofold expression difference in at least one pairwise comparison of the different conditions examined. Of those, one (NCU00628, hypothetical protein) shows an increase in the absence of AOD2 when grown in the presence of Cm (Figure 9A), three (NCU04392, NCU04752, NCU09144; all hypothetical proteins) have increases in the absence of AOD5 in the absence of Cm (Figure 9B), and NCU10051 (flavohemoglobin) shows an increase in expression in the presence of Cm when either AOD2 or AOD5 is absent (Figure 9C). A single gene (NCU02128, D-arabinitol dehydrogenase) showed decreased expression in the absence of AOD2 (Figure 9D), while two (NCU04899, malate dehydrogenase and NCU05627, a glucose transporter) showed decreased expression in the absence of either AOD2 or AOD5 (Figure 9E). One gene (NCU02623, mitochondrial hypoxia response) shows only increased expression in the presence of Cm, but no effects due to presence or absence of AOD2 or AOD5 (Figure 9F). Interestingly, the gene that showed a decrease in expression in the absence of AOD2 but was not affected by loss of AOD5 (NCU02128) (Figure 9D) has a peak profile that suggests considerable binding by AOD2, but very little, if any, AOD5 (see AOD2 binds to a repeat sequence in the genome).

**Table 4:** Genes examined from other overlapping positions on the Venn diagram

Gene(s) in Correct Orientation Relative to Peaks	qPCR Result*^a^*	AIM Sequence*^b^*	AIM Summit*^c^*	Group on Venn Diagram
NCU00628 (hypo)	2−	Y	N	AOD5 + and - Cm
NCU00629 (6-phosphofructokinase)	No effect	Y	N		
NCU01053 (hypo)	No effect	N	Na	AOD2 + and −Cm
NCU02128 (D-arabinitol dehydrogenase)	2+	Y	N	FN1/2+3/8+4/5
NCU02623 (mt hypoxia resp domain)	No effect	Y	Y	AOD5 + and − Cm
NCU04392 (hypo)	5−	Y	N	FN1/2+3/8+4/5
NCU04752 (hypo)	5−	Y	Y	AOD5 + and − Cm
NCU04753 (complex I protein)	No effect	Y	Y	
NCU04797 (FBP)	No effect	Y	Y	AOD5 + and − Cm
NCU04899 (malate dehydrogenase)	2+, 5+	N	Na	AOD5 + and − Cm
NCU05627 (high affinity glucose transporter	2+, 5+	N	Na	FN1/2+3/8+6/7
NCU05995 (ubiquitin)	No effect	Y	Y	FN3/8+4/5+6/7
NCU06211 (malate dehydrogenase)	No effect	Y	Y	FN1/2+3/8+6/7
NCU09144 (hypo)	5−	N*^d^*	Na	FN1/2+4/5+6/7
NCU10051 (flavohemoglobin)	2−, 5−	Y	Y	Not found*^e^*

Alternating gray and white rows indicate one or two genes in the correct orientation to a set of peaks found in the indicated Venn diagram position. NCU numbers are followed by known gene functions where applicable. hypo, hypothetical protein; Na, not applicable.

a Numbers indicate either AOD2 or AOD5. The plus sign indicates that protein is a positive regulator of the gene. The minus sign indicates that protein is a negative regulator of the gene.

b Y (yes), one or more AIM sequences present in upstream intergenic region of gene(s). N (no), no AIM sequence present in upstream intergenic region.

c Alignment of at least one AIM sequence at or near at least one ChIP-seq summit. Y, yes; N, no; Na, Not applicable.

d Associated with the 310 bp repeat sequence (see AOD2 binds to a repeat sequence in the genome).

e Gene not found in our Venn diagram where two or more groups overlap, but is found in the FN3/8 group alone. Examined as an example of a gene that was found in the *P. anserina* microarray study (Bovier *et al.* 2014).

### AOD2 binds to a repeat sequence in the genome

**Figure 10:**
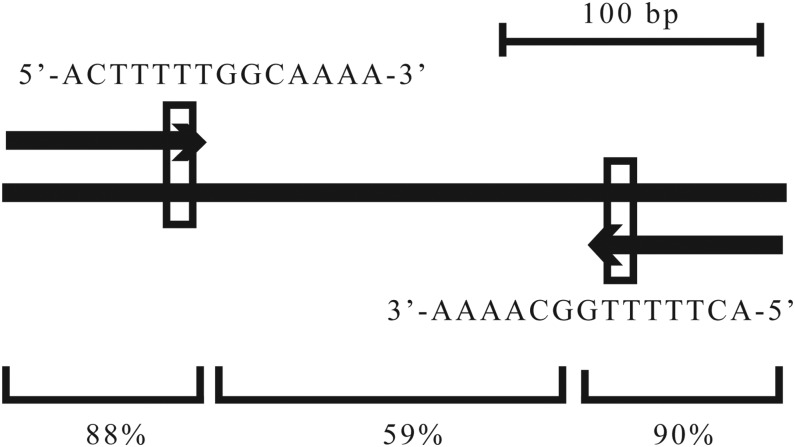
Representation of a repeated sequence element in the *N. crassa* genome that binds AOD2. The long black line represents the 310 bp repeat sequence. The arrows above and below the line represent the inverted repeats at the ends of the sequence. The small open boxes at the ends of the inverted repeat indicate the location of the highly conserved 14 bp sequence found associated with the AOD2-binding peak summits. The sequence of that site is shown. The thin lines at the bottom indicate the extent of the two inverted repeats compared with the rest of the sequence. Numbers give the percentage identity in the indicated region among all 11 of the 310 bp repeat sequences identified.

The upstream regions of the genes listed in Table 2 and Table 3 were analyzed for the existence of common sequence motifs in addition to the AIM sequence. A subset of genes was associated with another well conserved upstream sequence of 14 bases. Further investigation, based on searches and alignments of the *N. crassa* genome with the regions surrounding the 14 bp sequence revealed that 10 peak sets were associated with a 310 bp sequence that is repeated in 11 places throughout the genome with >85% identity (Table 5). There are also smaller subsets of the 310 bp sequence abundant in the genome. Each of the 310 bp sequences contains a set of 78 bp inverted repeats at the ends of the sequence (Figure 10 and Figure S5) that contain the originally identified 14 bp sequence. The inverted repeats may have functional significance since they are more highly conserved than the region between them (Figure 10).

**Table 5:** Sites in the *N. crassa* genome identified by BLASTN that have >75% identity to the 310 bp region upstream of NCU02128

Location	% Identity to NCU02128 Upstream Region	Gene(s) Surrounding Peaks
Supercontig 1	100	NCU02128 (D-arabinitol dehydrogenase)
1155455–1155765		
Supercontig 1	88	NCU01957*^a^* (mating type *A*/*a* related, AR2)
1865502–1865814		NCU01958*^a^* (mating type protein A-1)
Supercontig 1	87	NCU09144 (hypothetical protein)
5481346–5481655		
Supercontig 2	100	NCU03408 (hypothetical protein)
825079–825389		NCU03409 (RNA lariat debranching enzyme)
Supercontig 4	87	NCU04986 (hypothetical protein)
1096849–1097159		NCU11067 (hypothetical protein)
Supercontig 4	100	NCU07676 (hypothetical protein)
2271671–2271981		NCU07678*^b^* (hypothetical protein)
Supercontig 4	87	NCU05202 (hypothetical protein)
5768811–5769121		NCU05203 (hypothetical protein)
Supercontig 4	86	NCU04392 (hypothetical protein)
3512618–3512927		
Supercontig 5	87	NCU01321 (high affinity Ni transporter)
3654597–3654907		NCU01322 (transcription factor TFIIE complex α subunit)
Supercontig 6	87	NCU05527 (hypothetical protein)
1613020–1613330		
Supercontig 7	86	NCU09993 (helicase swr-1)
3974291–3974600*^c^*		NCU09460 (NADH:ubiquinone oxidoreductase 20.1 kD subunit)

Sites are shown in sequence according to the supercontig chromosome on which they occur.

a Both genes are in incorrect orientation relative to the peaks.

b Peaks much closer to the 5′ end of this gene of the pair.

c No ChIP-seq peaks seen associated with this locus.

Repeated sequences in the *Neurospora* genome are subject to a mutagenic process termed repeat induced point mutation (RIP) (Selker 1990). Although repeated sequences <0.4 kb are not normally considered good substrates for RIP (Watters *et al.* 1999), it has recently been shown that smaller sequences are affected by the process (Gladyshev and Kleckner 2014). It appears that the repeat units identified in this study have been subject to RIP since the majority of the changes found among the sequences are C:G to T:A transition mutations, which are characteristic of the process (Selker 1990). Of the 80 positions containing a change in at least one of the 11 sequences, only 15 are transversions, one is a deletion of a single base pair, one is an insertion of 2 bp, and 63 are transitions (Figure S5).

A characteristic of the peak sets associated with this repeat is the binding of relatively large amounts of AOD2 and smaller variable amounts of AOD5 (Figure S6), as compared with most “typical” peak sets observed in this study, such as for NCU07953 (AOD1) and NCU06940 (hypothetical protein) (Figure 5). One obvious exception to this rule is the repeat sequence in the region between genes NCU09460 and NCU09993, where no binding of either protein was observed (Figure S6). The variable amount of AOD5 bound at the other repeat sites is also shown by the fact that five of these peak sets were identified in the original group of 70 that had greater than fourfold enrichment in all four experiments. Another characteristic of the 10 sites that bind predominantly AOD2 is that the AOD2 peak is usually divided into two summits. The AOD2 peak summits at these sequences are always found associated with all or a portion of the highly conserved 14 bp sequence (Figure 10) that occurs near the ends of the inverted repeats. These AOD2-binding peaks are located in the correct upstream position for at least one of the genes adjacent to the peaks in nine of the 10 cases (Table 5).

The qPCR analyses in strains lacking AOD2 or AOD5 that were performed during the investigation of genes described above, included eight genes associated with the nine peak sets occurring in the repeated regions that have at least one gene in the proper orientation. Genes NCU03409, NCU05202, and NCU05203 showed no changes greater than twofold (Figure S2). NCU02128 was found to depend on AOD2 but not AOD5 for expression (Figure 9D), though the expression levels changed only two to threefold in the absence of AOD2. For NCU07678, no change in expression that depended on AOD2 or AOD5 was observed, but a small change in response to Cm was seen (Figure 6D). The greatest changes were seen for NCU03408, where both AOD2 and AOD5 were required for induction (Figure 6A). A substantial amount of AOD5 is bound at this peak set (Figure S6), and it was identified in the group of 65 peaks common to all four experiments. An AIM sequence is also associated with the summit where AOD5 is most abundant (compare Figure S3 and Figure S6). For NCU04392 and NCU09144, small increases in transcription were seen in strains lacking AOD5 (Figure 9B). The promoters of these two genes appear to bind very little, if any AOD5 (Figure S6), and it is difficult to imagine a negative regulatory role for AOD5 at these promoters. An alternative explanation might be that if AOD5 is not present, more AOD2 is available for binding and affecting transcription.

Visual inspection of the ChIP-seq data also revealed two additional peak sets (NCU01053 and NCU01224/NCU01225) with an excess of AOD2 binding over AOD5, as compared with the more typical peak sets. These genes are not associated with the repeat sequence, they do not have the conserved 14 bp sequence in their upstream regions, and they do not exhibit the “double summit” pattern of AOD2 binding seen at the repeat sites (Figure S7). Furthermore, no AIM sequences were found to be associated with these peak sets. All three of these genes were examined by qPCR in the strains lacking AOD2 or AOD5, and no changes greater than twofold were observed (Figure S2 and Figure S4).

## Discussion

In *N. crassa*, expression of AOX is greatly increased when the function of the sETC is perturbed by mutations or when chemicals inhibit the proper function of the chain. The induction occurs at the level of transcription and is dependent on the zinc cluster transcription factors AOD2 and AOD5 (Chae *et al.* 2007b; Tanton *et al.* 2003). In the current study, we have shown that AOD2 and AOD5 are localized to the nucleus and bound to the region upstream of genes, regardless of whether cells are grown under conditions that are noninducing or inducing for AOX expression. Thus, it appears that their activation is achieved by communication of an unknown signal(s) to the nucleus when cells are exposed to specific conditions that affect mitochondrial function. We previously showed that AOD2 and AOD5 form a heterodimer *in vitro* and now present evidence that this is also the case *in vivo*, though it is not known what fraction of the population of each protein exists in this state.

The orthologs of AOD2 and AOD5 in *A. nidulans* and *P. anserina* have been shown to control expression of the transcripts for the gluconeogenic enzymes PEPCK and FBP, as well as AOX (Suzuki *et al.* 2012; Bovier *et al.* 2014). Fluorescence microscopy showed that GFP-tagged orthologs of the proteins in *A. nidulans* localized to the nucleus regardless of whether cells were grown in glucose or a gluconeogenic carbon source (Suzuki *et al.* 2012). Here, we have shown that the expression of *N. crassa* PEPCK and FBP are increased following growth in gluconeogenic carbon sources. However, we found that only the expression of PEPCK was dependent on AOD2 (and presumably AOD5, though this was not directly tested), while that of FBP was not.

Our analysis of cultures grown in medium with different carbon sources revealed that the PEPCK mRNA was induced fourfold and sixfold by growth in acetate and ethanol as carbon sources, respectively, when compared with levels during growth with sucrose as the carbon source. On the other hand, growth in the presence of Cm had only a slight effect on PEPCK transcript levels, near the border of statistical significance. Conversely, the *aod-1* mRNA level was not significantly affected by growth in poor carbon sources, but was induced ∼18-fold by growth in the presence of Cm. Despite these differing patterns of expression for PEPCK and AOX, both inductions required AOD2 (and by implication, AOD5). Similar observations were made in *P. anserina* where the expression of AOX and several other genes was seen to be much higher during growth in the presence of antimycin A compared with when cultures were grown using acetate as a carbon source. However, the opposite was observed for the expression of the genes encoding PEPCK and FBP (Bovier *et al.* 2014). A related observation was also made in *A. nidulans* where mutations in the PAS domains of the AOD2 and AOD5 orthologs led to antimycin A sensitivity, but had only slight effects on ability to utilize gluconeogenic carbon sources (Suzuki *et al.* 2012). Various mechanisms are conceivable that might allow different signals to activate the AOD2/AOD5 heterodimer. For example, different signaling molecules might affect one protein and not the other, resulting in differential activation of transcription at specific sites due to surrounding sequence or additional protein factors.

Unexpectedly, we noted a difference in inducibility of the PEPCK transcript during growth in the presence of Cm with sucrose as the carbon source, depending on the growth medium used: about twofold (near the border of statistical significance) in low nitrogen-containing medium, but sevenfold in nitrogen-rich Vogel’s medium. Similarly, Cm induced AOX 18-fold in low nitrogen-containing medium, but ∼30-fold in Vogel’s medium. This suggests that the general nutritional status of a culture may also influence the regulation. Taken together, these data strongly suggest that different signals arising from various conditions of growth can differentially affect the transcription of a number of genes in an AOD2- and AOD5-dependent fashion. Whether the interpretation of different signals can be achieved by AOD2 and AOD5 alone, or if additional factors are required, is not known.

The growth analysis also revealed that deletion mutants of *aod-2* and *aod-5* grew at a reduced rate compared with wild-type cells, even when sucrose was the carbon source. This suggested that the proteins may play roles in regulating other pathways, in addition to AOX and gluconeogenesis. The results of the ChIP-seq experiments, where a large number of peaks were identified by MACS2 analysis in each of the four experiments, supported this idea. As described in the *Results*, we chose to focus on peak sets that fulfilled various criteria, leaving 98 genes as candidates for being controlled by AOD2 and AOD5. Applying these criteria may result in the exclusion of some genes that are legitimate targets, but should help minimize the number of false positives. Inspection of the peaks revealed a wide array of binding characteristics with respect to size and shape of peaks. This may reflect different binding efficiencies which could be due to sequence variation, involvement of additional binding factors, or state of chromatin in different regions of the growing mycelium.

Thirty-three of these 98 genes were analyzed by qPCR in both mutant and control backgrounds in an attempt to validate the role of the proteins as transcription factors for the identified genes. Eleven of the genes chosen for qPCR validation, including *aod-1*, were regulated in a positive fashion that required both AOD2 and AOD5 (Figure 6, A and B). However, even among these 11, additional complexities were apparent. For example, eight of the genes demonstrated an obvious response to Cm, while three others did not. Furthermore, for most of those that did, a response to Cm was retained, even in the absence of AOD2 and AOD5, though the overall levels of induction were much lower. Thus, it appears that the promoters of some genes are influenced by both the AOD2/AOD5 heterodimer, as well as other factors that interpret additional signals generated from growth in the presence of Cm. The other three positively controlled genes appear to lack this extra involvement with Cm. These different responses further support the notion that other factors may be involved in the regulation of these genes. Validation by qPCR also revealed that three genes were negatively controlled by AOD2 and/or AOD5, since loss of the proteins resulted in increased transcript levels (Figure 6C).

The AIM has been shown to be within the promoter region of genes controlled by AOD2/AOD5 or their orthologs (Chae *et al.* 2007a; Suzuki *et al.* 2012; Bovier *et al.* 2014). The ChIP-seq peaks for 10 of the 11 genes that are positively controlled by the two transcription factors have at least one AIM sequence aligned with at least one summit. The exception is NCU03408. Here, an AIM sequence aligns reasonably well with the AOD5 summits, but not with the largest AOD2 summits. Interestingly, this is a gene associated with the repeat sequence for binding AOD2, and the largest AOD2 summits align with the conserved consensus sequence found within the repeat. Thus, at this site, it appears that a standard AIM and an AOD2-specific binding site occur in close proximity. Of the three negatively regulated genes, NCU04874 (AOD3) and NCU06230 (Ser/Thr kinase) have an AIM sequence at the peak summits, while NCU03749 (hydroxyacyl glutathione hydrolase) does not.

An additional five genes examined by qPCR showed no effect on transcript levels when AOD2 or AOD5 were missing, but did show increased transcript levels in the presence of Cm (Figure 6D). The fact that the promoters of these genes bind the two transcription factors but show no effects when the factors are missing under the conditions examined, again suggests that other factors may play a role in inducing transcription. In these cases, the role, if any, of AOD2 and AOD5 is unclear. However, the homolog of one of these five genes (NCU11414) was also identified in the *P. anserina* microarray study (Bovier *et al.* 2014) as being upregulated by gain-of-function mutations in the orthologs of AOD2 and AOD5 (RSE2 and RSE3, respectively) and downregulated in strains with deletions of the genes. These findings support a role for AOD2 and AOD5 in the regulation of NCU11414. The relationship identified for two other genes in this category (NCU02474 and NCU08946) with their adjacent, divergently transcribed genes (see *Results*), points to a complex mechanism of transcriptional control involving AOD2, AOD5, other factors, and possibly different signals resulting from growth in the presence of Cm.

Many genes examined during our validation process failed to show differences in expression in the absence of either protein or in the presence or absence of Cm. Some of these may simply represent false positives, which can be frequent in ChIP-seq studies (Worsley Hunt and Wasserman 2014; Jain *et al.* 2015). On the other hand, many such genes show obvious binding of both proteins in their upstream regions. For example, both NCU04307 (MSF1, mitochondrial tRNA synthetase Phe) and NCU07807 (fructose bisphosphate aldolase) are the only genes in the correct orientation relative to the peaks upstream of their coding sequences. In both cases, the peaks from all four experiments are robust and include an AIM sequence at their summit. It is conceivable that these genes respond in an AOD2/AOD5-dependent manner to other induction signals arising from different stresses. As discussed above, this might also require the action of other proteins.

A relevant question with respect to AOD2 and AOD5, is whether they can act independently of each other as monomers, homodimers, or dimers with other transcription factors. Such interactions, including interactions with other classes of transcription factor, have been described for zinc cluster proteins (MacPherson *et al.* 2006), The Oaf1 zinc cluster protein of *S. cerevisiae* is known to function both alone or as part of a heterodimer with another zinc cluster protein, Pip2 (Karpichev and Small 1998; Baumgartner *et al.* 1999). Furthermore, it has been observed in *P. anserina* that loss of RSE3 (the AOD5 ortholog), but not RSE2 (the AOD2 ortholog), results in reduced fertility (Bovier *et al.* 2014), implying that RSE3 has at least one function that is independent of RSE2.

ChIP-seq data suggesting robust binding of one protein without the other could be taken as evidence that binding to DNA as independent proteins is possible. Although we cannot directly compare ChIP-seq peak heights obtained with different antibodies, it is apparent from visual inspection of the data that the majority of peaks reflect binding of both proteins. However, it was also apparent that in certain cases the ratios of the two proteins were considerably skewed compared with the typical peak sets observed. While we cannot rule out the possibility that binding of additional proteins at certain promoters may mask the epitope tag of one protein but not the other, it is also possible that the proteins bind without the other at certain sites. For AOD5, the peaks associated with a few genes seem to bind substantially more AOD5 than AOD2; perhaps the best example is NCU06211 (malate dehydrogenase). However, no influence of either AOD2 or AOD5 on transcript levels of the gene could be seen during growth in standard conditions or in the presence of Cm.

Twelve peak sets were identified as binding larger amounts of AOD2 compared with AOD5. Two of these sets had no obvious defining characteristics. However, 10 of the sets were associated with the 310 bp repeat region. In nine of these 10 cases, the repeat region is in a position that corresponds to the upstream region of one or both of the flanking genes. Seven of these genes were examined by qPCR for effects on expression due to Cm and loss of AOD2 or AOD5. A variety of effects were observed so that for the conditions tested it cannot be concluded that AOD2 is required for expression of genes at all these sites. Only one of the 11 repeated regions with >75% identity found in the genome was not associated with AOD2 binding. It is possible that the arrangement of chromatin, or other bound proteins at this locus under the conditions tested, prevents access for AOD2.

The origin of the 310 bp repeat regions and the relevance of AOD2 binding within them are not apparent. BLAST searches of other *Neurospora* species available at FungiDB (http://fungidb.org/fungidb/) revealed that *N. tetrasperma* contained four sequences with high identity to the entire 310 bp region. *N. discreta* had no sites related to the entire 310 bp sequence but did have two smaller sites with high similarity to portions of the inverted repeat region. Thus, the sequence may have originated in an ancestral species, but is only being maintained in *N. crassa*. A consensus sequence was identified at the summit of the AOD2 binding sites, but further work is required to determine if it is actually a binding site for the protein.

Analysis of genes by FunCat showed clear enrichment for genes in certain categories. Within our set of 98 genes associated with the 65 peak sets, several are involved with respiration or energy metabolism. In addition, we know of at least one additional gene (NCU08674) in our data set that is categorized simply as a pentatricopeptide repeat protein in the database, but has now been shown to be involved in the assembly of respiratory complexes in *N. crassa* (Solotoff *et al.* 2015). Consideration of the number and type of genes affected by RSE2 and RSE3 in the *P. anserina* microarray study led to the suggestion that the transcription factors are involved in strategies that contribute to the defense and adaptation of the organism (Bovier *et al.* 2014).

Other categories identified by FunCat analysis in the current study involved metabolism of glutamate and glycine. Glutamate, or the related amino acid glutamine, provide the initial nitrogen source for the synthesis of other amino acids, purines, and pyrimidines. Glutamate is typically formed by amination of the TCA cycle intermediate α-ketoglutarate (Davis and Wong 2010; Magasanik and Kaiser 2002). Interestingly, when mitochondrial function is low in *S. cerevisiae*, the production of enzymes necessary for the synthesis of α-ketoglutarate are under the control of the classic retrograde response (RTG) system originally discovered in this organism. Thus, *rtg* mutants in *S. cerevisiae* are glutamate auxotrophs in conditions where respiratory activity is low (Liu and Butow 1999, 2006; Liao and Butow 1993). In the current study, we observed no evidence of the involvement of AOD2 and AOD5 in the regulation of genes for α-ketoglutarate synthesis, but the upstream region of five genes involved in glutamate metabolism were found to be associated with AOD2/AOD5 binding.

The glycine enzymes identified in the current study are named for the glycine cleavage system, but the system is also known as glycine synthase and can act in the reverse direction to synthesize glycine (Kikuchi and Hiraga 1982). The complex consists of four proteins housed in the mitochondrion. These proteins convert glycine to CO_2_ and NH_3_, while also forming the 1-carbon donor molecule, 5,10-methylenetetrahydrofolate. FunCat analysis revealed that three (NCU02475, NCU06424, and NCU08877) of the four genes encoding these proteins were present in the group of 65 peaks common to all four ChIP-seq experiments. The peaks associated with NCU02475 were quite robust and qPCR analysis confirmed that AOD2 and AOD5 were required for optimal expression. Peak sets associated with the other two genes were very small. Expression of NCU08877 was not affected by the absence of AOD2 or AOD5. NCU06424 expression was not examined. However, all three sets of peaks were associated with AIM sequences at their summits. Both glutamate and glycine are required for the synthesis of glutathione. Glutathione is an important molecule that provides a response to various cellular stresses, including reactive oxygen species in mitochondria (Forman *et al.* 2009; Ribas *et al.* 2014). Since these damaging species may increase when the sETC is impaired, it is conceivable that AOD2/AOD5 may be involved in increasing production of at least some of the enzymes required for synthesis of these two amino acids, so that substrates for glutathione synthesis are available during oxidative stress.

It has been suggested that malate is an inducing molecule for the AOD2 and AOD5 orthologs in *A. nidulans* (Suzuki *et al.* 2012). Though not identified in our FunCat analysis, we noted that two genes associated with malate metabolism were included in our group of 65 peak sets (NCU03651, malic enzyme and NCU10007, malate synthase) and two others (NCU04899, mitochondrial malate dehydrogenase and NCU06211, cytosolic malate dehydrogenase) appeared among the group with good peak sets that did not fall within the group of 65 peak sets. NCU03651 and NCU10007 showed no response to Cm, but AOD2 and AOD5 were required for their expression both with and without Cm in the growth medium. On the other hand, NCU04899 showed a response to Cm that required the presence of both AOD2 and AOD5.

In conclusion, our data show an involvement of AOD2 and AOD5 in the expression of several genes that play a role in energy metabolism and various metabolic pathways. The factors appear to act predominantly in a positive fashion, but examples of repressing activity were also seen. The majority of genes appear to bind the transcription factors as a heterodimer, but rare cases of preferential binding of one or the other were also seen. The mechanism of function of the proteins is complex, allowing different responses to different conditions.

## Supplementary Material

Supplemental material is available online at www.g3journal.org/lookup/suppl/doi:10.1534/g3.116.035402/-/DC1.

## References

[bib1] Arthanari Y. Heintzen C. Griffiths-Jones S. Crosthwaite S. K., 2014 Natural antisense transcripts and long non-coding RNA in Neurospora crassa. PLoS One 9(3): e91353. 24621812 10.1371/journal.pone.0091353 PMC3951366

[bib2] Baumgartner U. Hamilton B. Piskacek M. Ruis H. Rottensteiner H., 1999 Functional analysis of the Zn(2)Cys(6) transcription factors Oaf1p and Pip2p. Different roles in fatty acid induction of beta-oxidation in Saccharomyces cerevisiae. J. Biol. Chem. 274(32): 22208–22216. 10428786 10.1074/jbc.274.32.22208

[bib3] Bertrand H. Argan A. Szakacs N. A., 1983 Genetic control of the biogenesis of cyanide insensitive respiration in *Neurospora crassa*, pp. 495–507 in *Mitochondria*, edited by Schweyen R. J. Wolf K. Kaudewitz F. Walter de Gruyter Co., Berlin.

[bib4] Bevan R. B. Lang B. F., 2004 Mitochondrial genome evolution: the origin of mitochondria and of eukaryotes, pp. 1–35 in *Mitochondrial Function and Biogenetics*, *Topics in Current Genetics*, Vol. 8, edited by Kohler C. Bauer M. F. Springer-Verlag, Berlin, Heidelberg.

[bib5] Borghouts C. Werner A. Elthon T. E. Osiewacz H. D., 2001 Copper-modulated gene expression and senescence in the filamentous fungus *Podospora anserina*. Mol. Cell. Biol. 21: 390–399. 11134328 10.1128/MCB.21.2.390-399.2001 PMC86578

[bib6] Bovier E. Sellem C. H. Humbert A. Sainsard-Chanet A., 2014 Genetic and functional investigation of Zn(2)Cys(6) transcription factors RSE2 and RSE3 in Podospora anserina. Eukaryot. Cell 13(1): 53–65. 24186951 10.1128/EC.00172-13 PMC3910949

[bib7] Burger G. Gray M. W. Forget L. Lang B. F., 2013 Strikingly bacteria-like and gene-rich mitochondrial genomes throughout jakobid protists. Genome Biol. Evol. 5(2): 418–438. 23335123 10.1093/gbe/evt008 PMC3590771

[bib8] Chae M. S. Nargang F. E., 2009 Investigation of regulatory factors required for alternative oxidase production in Neurospora crassa. Physiol. Plant. 137(4): 407–418. 19493307 10.1111/j.1399-3054.2009.01239.x

[bib9] Chae M. S. Lin C. C. Kessler K. E. Nargang C. E. Tanton L. L., 2007 a Identification of an alternative oxidase induction motif in the promoter region of the *aod-1* gene in *Neurospora crassa*. Genetics 175: 1597–1606. 17237510 10.1534/genetics.106.068635 PMC1855127

[bib10] Chae M. S. Nargang C. E. Cleary I. A. Lin C. C. Todd A. T., 2007 b Two zinc cluster transcription factors control induction of alternative oxidase in *Neurospora crassa*. Genetics 177: 1997–2006. 18073419 10.1534/genetics.107.078212 PMC2219509

[bib11] Chakravarty A. Carlson J. M. Khetani R. S. Gross R. H., 2007 A novel ensemble learning method for de novo computational identification of DNA binding sites. BMC Bioinformatics 8: 249. 17626633 10.1186/1471-2105-8-249 PMC1950314

[bib12] da Cunha F. M. Torelli N. Q. Kowaltowski A. J., 2015 Mitochondrial retrograde signaling: triggers, pathways, and outcomes. Oxid. Med. Cell. Longev. 2015: 482582. 26583058 10.1155/2015/482582 PMC4637108

[bib13] Davis M. A. Wong K. H., 2010 Nitrogen metabolism in filamentous fungi, pp. 325–338 in *Cellular and Molecular Biology of Filamentous Fungi*, edited by Borkovich K. A. Ebbole D. J. ASM Press, Washington, DC.

[bib14] Davis R. H. De Serres F. J., 1970 Genetic and microbiological research techniques for *Neurospora crassa*. Methods Enzymol. 17: 79–143.

[bib15] Descheneau A. T. Cleary I. A. Nargang F. E., 2005 Genetic evidence for a regulatory pathway controlling alternative oxidase production in *Neurospora crassa*. Genetics 169: 123–135. 15466423 10.1534/genetics.104.034017 PMC1448880

[bib16] Dufour E. Boulay J. Rincheval V. Sainsard-Chanet A., 2000 A causal link between respiration and senescence in *Podospora anserina*. Proc. Natl. Acad. Sci. USA 97: 4138–4143. 10759557 10.1073/pnas.070501997 PMC18174

[bib17] Edwards D. L. Chalmers J. H. Jr Guzik H. J. Warden J. T., 1976 Assembly of the cyanide-insensitive respiratory pathway in Neurospora crassa, pp. 865–872 in Genetics and Biogenesis of Chloroplasts and Mitochondria, edited by Bucher T. H. Neupert W. Sebald W. Werner S. Elsevier/North-Holland Biomedical Press, Amsterdam.

[bib18] Elstner M. Andreoli C. Klopstock T. Meitinger T. Prokisch H., 2009 The mitochondrial proteome database: MitoP2. Methods Enzymol. 457: 3–20. 19426859 10.1016/S0076-6879(09)05001-0

[bib19] Forman H. J. Zhang H. Rinna A., 2009 Glutathione: overview of its protective roles, measurement, and biosynthesis. Mol. Aspects Med. 30(1–2): 1–12. 18796312 10.1016/j.mam.2008.08.006 PMC2696075

[bib20] Gladyshev E. Kleckner N., 2014 Direct recognition of homology between double helices of DNA in Neurospora crassa. Nat. Commun. 5: 3509. 24699390 10.1038/ncomms4509 PMC4000310

[bib21] Good A. G. Crosby W. L., 1989 Anaerobic induction of alanine aminotransferase in barley root tissue. Plant Physiol. 90: 1305–1309. 16666927 10.1104/pp.90.4.1305 PMC1061887

[bib22] Guo J. Huang G. Cha J. Liu Y., 2010 Biochemical methods used to study the gene expression and protein complexes in the filamentous fungus Neurospora crassa. Methods Mol. Biol. 638: 189–200. 20238270 10.1007/978-1-60761-611-5_14

[bib23] Hoppins S. C. Go N. E. Klein A. Schmitt S. Neupert W., 2007 Alternative splicing gives rise to different isoforms of the *Neurospora crassa* Tob55 protein that vary in their ability to insert β−barrel proteins into the outer mitochondrial membrane. Genetics 177: 137–149. 17660559 10.1534/genetics.107.075051 PMC2013688

[bib24] Huh W. Kang S., 2001 Characterization of the gene family encoding alternative oxidase from *Candida albicans*. Biochem. J. 356: 595–604. 11368790 10.1042/0264-6021:3560595 PMC1221874

[bib25] Jain D. Baldi S. Zabel A. Straub T. Becker P. B., 2015 Active promoters give rise to false positive ‘Phantom Peaks’ in ChIP-seq experiments. Nucleic Acids Res. 43(14): 6959–6968. 26117547 10.1093/nar/gkv637 PMC4538825

[bib26] Jazwinski S. M., 2013 The retrograde response: when mitochondrial quality control is not enough. Biochim. Biophys. Acta 1833(2): 400–409. 22374136 10.1016/j.bbamcr.2012.02.010 PMC3389569

[bib27] Karpichev I. V. Small G. M., 1998 Global regulatory functions of Oaf1p and Pip2p (Oaf2p), transcription factors that regulate genes encoding peroxisomal proteins in Saccharomyces cerevisiae. Mol. Cell. Biol. 18(11): 6560–6570. 9774671 10.1128/mcb.18.11.6560 PMC109241

[bib28] Kikuchi G. Hiraga K., 1982 The mitochondrial glycine cleavage system. Unique features of the glycine decarboxylation. Mol. Cell. Biochem. 45(3): 137–149. 6750353 10.1007/BF00230082

[bib29] Kirimura K. Matsui T. Sugano S. Usami S., 1996 Enhancement and repression of cyanide-insensitive respiration in *Aspergillus niger*. FEMS Microbiol. Lett. 141: 251–254. 8768530 10.1111/j.1574-6968.1996.tb08393.x

[bib30] Kramer C. Loros J. J. Dunlap J. C. Crosthwaite S. K., 2003 Role for antisense RNA in regulating circadian clock function in Neurospora crassa. Nature 421(6926): 948–952. 12607002 10.1038/nature01427

[bib31] Laemmli U. K., 1970 Cleavage of structural proteins during the assembly of the head of bacteriophage T4. Nature 227: 680–685. 5432063 10.1038/227680a0

[bib32] Lambowitz A. M., 1979 Preparation and analysis of mitochondrial ribosomes. Methods Enzymol. 54: 421–433. 10.1016/0076-6879(79)59103-4 440084

[bib33] Lenaz G. Genova M. L., 2009 Structural and functional organization of the mitochondrial respiratory chain: a dynamic super-assembly. Int. J. Biochem. Cell Biol. 41(10): 1750–1772. 19711505 10.1016/j.biocel.2009.04.003

[bib34] Li H. Durbin R., 2009 Fast and accurate short read alignment with Burrows-Wheeler transform. Bioinformatics 25(14): 1754–1760. 19451168 10.1093/bioinformatics/btp324 PMC2705234

[bib35] Liao X. Butow R. A., 1993 RTG1 and RTG2: two yeast genes required for a novel path of communication from mitochondria to the nucleus. Cell 72: 61–71. 8422683 10.1016/0092-8674(93)90050-z

[bib36] Liu Z. Butow R. A., 1999 A transcriptional switch in the expression of yeast tricarboxylic acid cycle genes in response to a reduction or loss of respiratory function. Mol. Cell. Biol. 19(10): 6720–6728. 10490611 10.1128/mcb.19.10.6720 PMC84662

[bib37] Liu Z. Butow R. A., 2006 Mitochondrial retrograde regulation. Annu. Rev. Genet. 40: 159–185. 16771627 10.1146/annurev.genet.40.110405.090613

[bib38] MacPherson S. Larochelle M. Turcotte B., 2006 A fungal family of transcriptional regulators: the zinc cluster proteins. Microbiol. Mol. Biol. Rev. 70(3): 583–604. 16959962 10.1128/MMBR.00015-06 PMC1594591

[bib39] Magasanik B. Kaiser C. A., 2002 Nitrogen regulation in Saccharomyces cerevisiae. Gene 290(1–2): 1–18. 12062797 10.1016/s0378-1119(02)00558-9

[bib40] Marzluf G. A., 1997 Genetic regulation of nitrogen metabolism in the fungi. Microbiol. Mol. Biol. Rev. 61(1): 17–32. 9106362 10.1128/mmbr.61.1.17-32.1997 PMC232598

[bib41] McDonald A. E., 2008 Alternative oxidase: an inter-kingdom perspective on the function and regulation of this broadly distributed “cyanide-resistant” terminal oxidase. Funct. Plant Biol. 35: 535–552. 10.1071/FP08025 32688810

[bib42] McDonald A. E. Vanlerberghe G. C., 2006 Origins, evolutionary history, and taxonomic distribution of alternative oxidase and plastoquinol terminal oxidase. Comp. Biochem. Physiol. Part D 1: 357–364. 10.1016/j.cbd.2006.08.001 20483267

[bib43] McDonald A. E. Amirsadeghi S. Vanlerberghe G. C., 2003 Prokaryotic orthologues of mitochondrial alternative oxidase and plastid terminal oxidase. Plant Mol. Biol. 53(6): 865–876. 15082931 10.1023/B:PLAN.0000023669.79465.d2

[bib44] McDonald A. E. Vanlerberghe G. C. Staples J. F., 2009 Alternative oxidase in animals: unique characteristics and taxonomic distribution. J. Exp. Biol. 212(Pt 16): 2627–2634. 19648408 10.1242/jeb.032151

[bib45] Meisinger C. Sickmann A. Pfanner N., 2008 The mitochondrial proteome: from inventory to function. Cell 134(1): 22–24. 18614007 10.1016/j.cell.2008.06.043

[bib46] Metzenberg R. L., 2003 Vogel’s medium N salts: avoiding the need for ammonium nitrate. Fungal Genet. Newsl. 50: 14.

[bib47] Moore A. L. Shiba T. Young L. Harada S. Kita K., 2013 Unraveling the heater: new insights into the structure of the alternative oxidase. Annu. Rev. Plant Biol. 64: 637–663. 23638828 10.1146/annurev-arplant-042811-105432

[bib48] Nargang F. E. Rapaport D., 2007 Neurospora crassa as a model organism for mitochondrial biogenesis, pp. 107–123 in *Mitochondria. Practical Protocols*, edited by Leister D. L. Herrmann J. Humana Press, Totowa, NJ. 10.1007/978-1-59745-365-3_8 18314721

[bib49] Neimanis K. Staples J. F. Huner N. P. McDonald A. E., 2013 Identification, expression, and taxonomic distribution of alternative oxidases in non-angiosperm plants. Gene 526(2): 275–286. 23664893 10.1016/j.gene.2013.04.072

[bib50] Oliveros, J. C., 2007–2015 Venny. An interactive tool for comparing lists with Venn’s diagrams. Accessed June 23, 2015. Available at: http://bioinfogp.cnb.csic.es/tools/venny/index.html

[bib51] Priebe S. Kreisel C. Horn F. Guthke R. Linde J., 2015 FungiFun2: a comprehensive online resource for systematic analysis of gene lists from fungal species. Bioinformatics 31(3): 445–446. 25294921 10.1093/bioinformatics/btu627 PMC4308660

[bib52] Ribas V. Garcia-Ruiz C. Fernandez-Checa J. C., 2014 Glutathione and mitochondria. Front. Pharmacol. 5: 151. 25024695 10.3389/fphar.2014.00151 PMC4079069

[bib53] Ruepp A. Zollner A. Maier D. Albermann K. Hani J., 2004 The FunCat, a functional annotation scheme for systematic classification of proteins from whole genomes. Nucleic Acids Res. 32(18): 5539–5545. 15486203 10.1093/nar/gkh894 PMC524302

[bib54] Sakajo S. Minagawa N. Yoshimoto A., 1993 Characterization of the alternative oxidase protein in the yeast *Hansenula anomala*. FEBS 318: 310–312. 10.1016/0014-5793(93)80535-3 8440388

[bib55] Schmitt S. Ahting U. Eichacker L. Granvogl B. Go N. E., 2005 Role of Tom5 in maintaining the structural stability of the TOM complex of mitochondria. J. Biol. Chem. 280: 14499–14506. 15701639 10.1074/jbc.M413667200

[bib56] Selker E. U., 1990 Premeiotic instability of repeated sequences in *Neurospora crassa*. Annu. Rev. Genet. 24: 579–613. 2150906 10.1146/annurev.ge.24.120190.003051

[bib57] Sellem C. H. Bovier E. Lorin S. Sainsard-Chanet A., 2009 Mutations in two zinc cluster proteins activate alternative respiratory and gluconeogenic pathways and restore senescence in long-lived respiratory mutants of *Podospora anserina*. Genetics 182: 69–78. 19255367 10.1534/genetics.109.100834 PMC2674842

[bib58] Sherman E. L. Go N. E. Nargang F. E., 2005 Functions of the small proteins in the TOM complex of *Neurospora crassa*. Mol. Biol. Cell 16: 4172–4182. 15987740 10.1091/mbc.E05-03-0187 PMC1196328

[bib59] Shi N.-Q. Davis B. Sherman F. Cruz J. Jeffries T. W., 1999 Disruption of the cytochrome c gene in xylose-utilizing yeast *Pichia stipitis* leads to higher ethanol production. Yeast 15: 1021–1030. 10455226 10.1002/(SICI)1097-0061(199908)15:11<1021::AID-YEA429>3.0.CO;2-V

[bib60] Solotoff V. Moseler R. Schulte U., 2015 Two pentatricopeptide repeat domain proteins are required for the synthesis of respiratory complex I. Curr. Genet. 61(1): 19–29. 25108509 10.1007/s00294-014-0441-2

[bib61] Staben C. Jensen B. Singer M. Pollock J. Schechtman M., 1989 Use of bacterial hygromycin B resistance gene as a dominant selectable marker in *Neurospora crassa* transformation. Fungal Genet. Newsl. 36: 79–81.

[bib62] Stein L. D. Mungall C. Shu S. Caudy M. Mangone M., 2002 The generic genome browser: a building block for a model organism system database. Genome Res. 12(10): 1599–1610. 12368253 10.1101/gr.403602 PMC187535

[bib63] Suzuki Y. Murray S. L. Wong K. H. Davis M. A. Hynes M. J., 2012 Reprogramming of carbon metabolism by the transcriptional activators AcuK and AcuM in Aspergillus nidulans. Mol. Microbiol. 84(5): 942–964. 22500966 10.1111/j.1365-2958.2012.08067.x

[bib64] Talbot K. J. Russell P. J., 1982 Nuclear buoyant density determination and the purification and characterization of wild-type neurospora nuclei using percoll density gradients. Plant Physiol. 70(3): 704–708. 16662561 10.1104/pp.70.3.704 PMC1065756

[bib65] Tanton L. L. Nargang C. E. Kessler K. E. Li Q. Nargang F. E., 2003 Alternative oxidase expression in *Neurospora crassa*. Fungal Genet. Biol. 39: 176–190. 12781676 10.1016/s1087-1845(03)00002-1

[bib66] Tissieres A. Mitchell H. K. Haskins F. A., 1953 Studies on the respiratory system of the poky strain of *Neurospora*. J. Biol. Chem. 205: 423–433. 13117920

[bib67] Watters M. K. Randall T. A. Margolin B. S. Selker E. U. Stadler D. R., 1999 Action of repeat-induced point mutation on both strands of a duplex and on tandem duplications of various sizes in *Neurospora*. Genetics 153(2): 705–714. 10511550 10.1093/genetics/153.2.705 PMC1460768

[bib68] Worsley Hunt R. Wasserman W. W., 2014 Non-targeted transcription factors motifs are a systemic component of ChIP-seq datasets. Genome Biol. 15(7): 412. 25070602 10.1186/s13059-014-0412-4 PMC4165360

[bib69] Yukioka H. Inagaki S. Tanaka R. Katoh K. Miki N., 1998 Transcriptional activation of the alternative oxidase gene of the fungus *Magnaporthe grisea* by a respiratory-inhibiting fungicide and hydrogen peroxide. Biochim. Biophys. Acta 1442: 161–169. 9804939 10.1016/s0167-4781(98)00159-6

[bib70] Zhang Y. Liu T. Meyer C. A. Eeckhoute J. Johnson D. S., 2008 Model-based analysis of ChIP-Seq (MACS). Genome Biol. 9(9): R137. 18798982 10.1186/gb-2008-9-9-r137 PMC2592715

