# Supplementary material for: Alternative Oxidase Transcription Factors AOD2 and AOD5 of Neurospora crassa Control the Expression of Genes Involved in Energy Production and Metabolism
Source: G3 (Bethesda). 2016 Dec 16;7(2):449–66. doi: 10.1534/g3.116.035402 (PMC5295593; doi:10.1534/g3.116.035402)
Supplement: Supplementary file 4 [file 449FigureS2.docx]

Figure S2. Genes from the common set of 65 ChIP-seq peaks that showed no effect on transcript levels due to loss of AOD2, AOD5, or growth in Cm. (.ai, 1.24 MB)

[http://www.g3journal.org/lookup/suppl/doi:10.1534/g3.116.035402/-/DC1/FigureS2.ai](http://www.g3journal.org/lookup/suppl/doi:10.1534/g3.116.035402/-/DC1/FigureS1.ai)
