# Supplementary material for: Alternative Oxidase Transcription Factors AOD2 and AOD5 of Neurospora crassa Control the Expression of Genes Involved in Energy Production and Metabolism
Source: G3 (Bethesda). 2016 Dec 16;7(2):449–66. doi: 10.1534/g3.116.035402 (PMC5295593; doi:10.1534/g3.116.035402)
Supplement: Supplementary file 5 [file 449FigureS3.pdf]

Figure S3 page 1

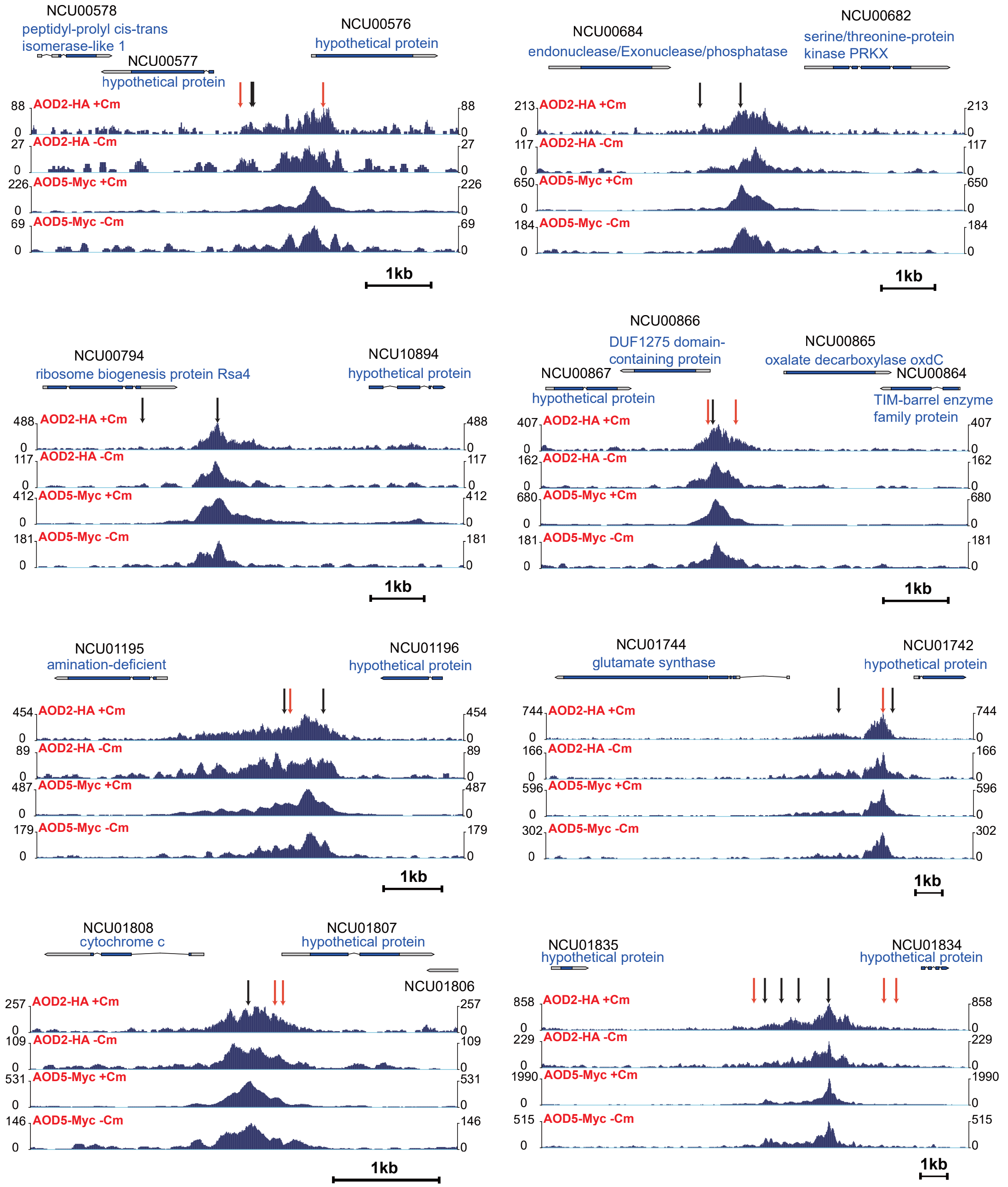

Figure S3 page 2

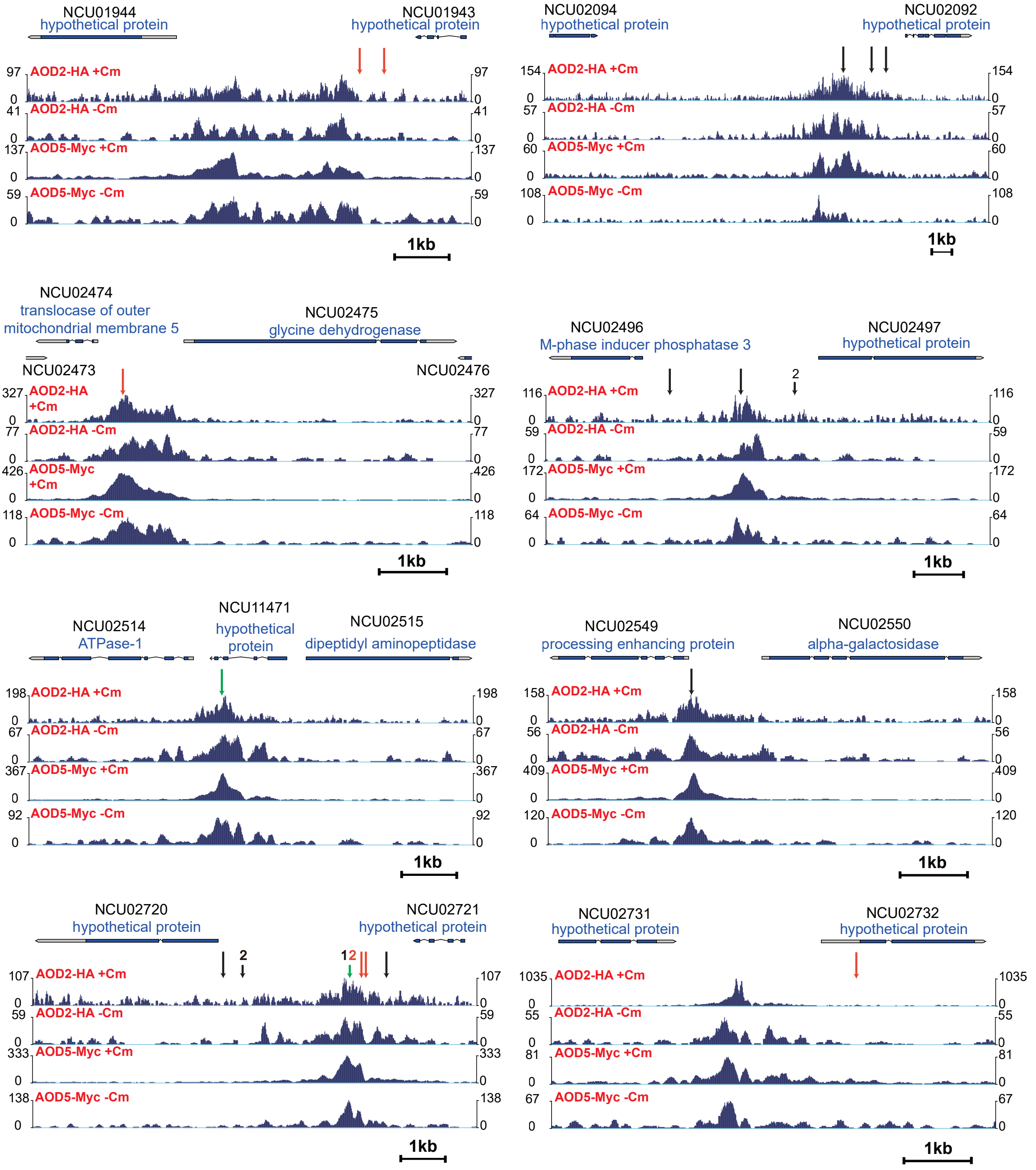

Figure S3 page 3

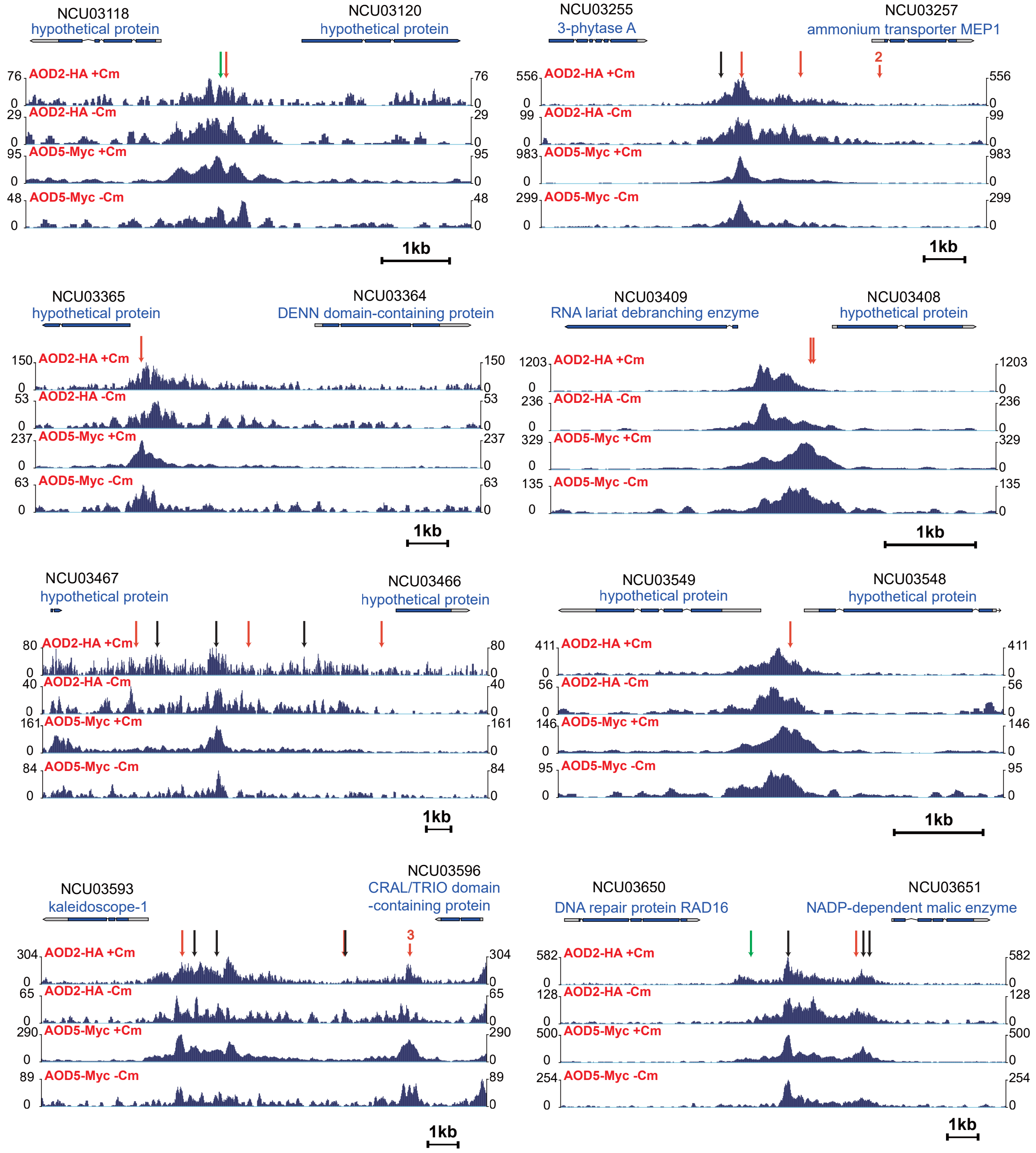

Figure S3 page 4

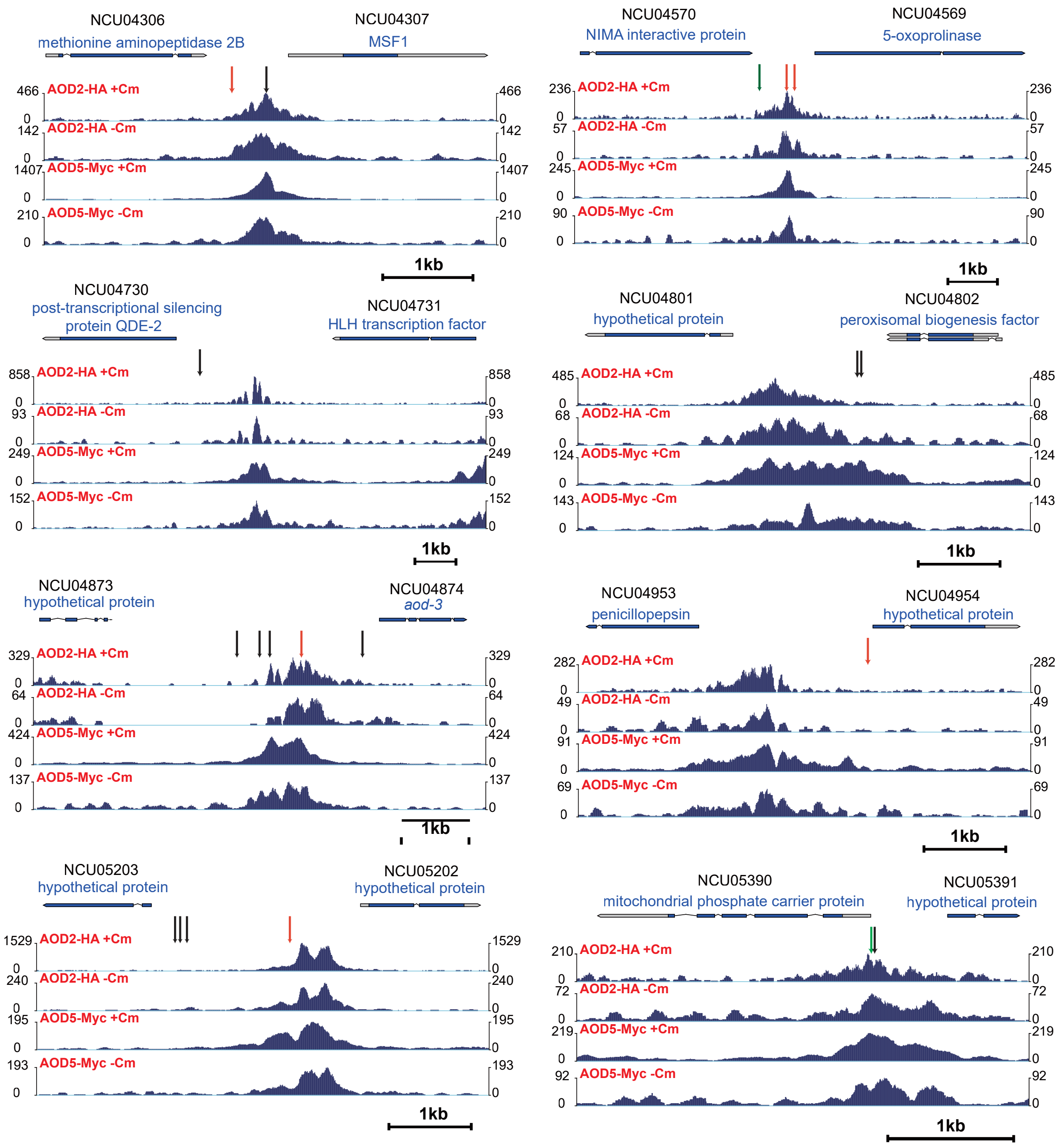

Figure S3 page 5

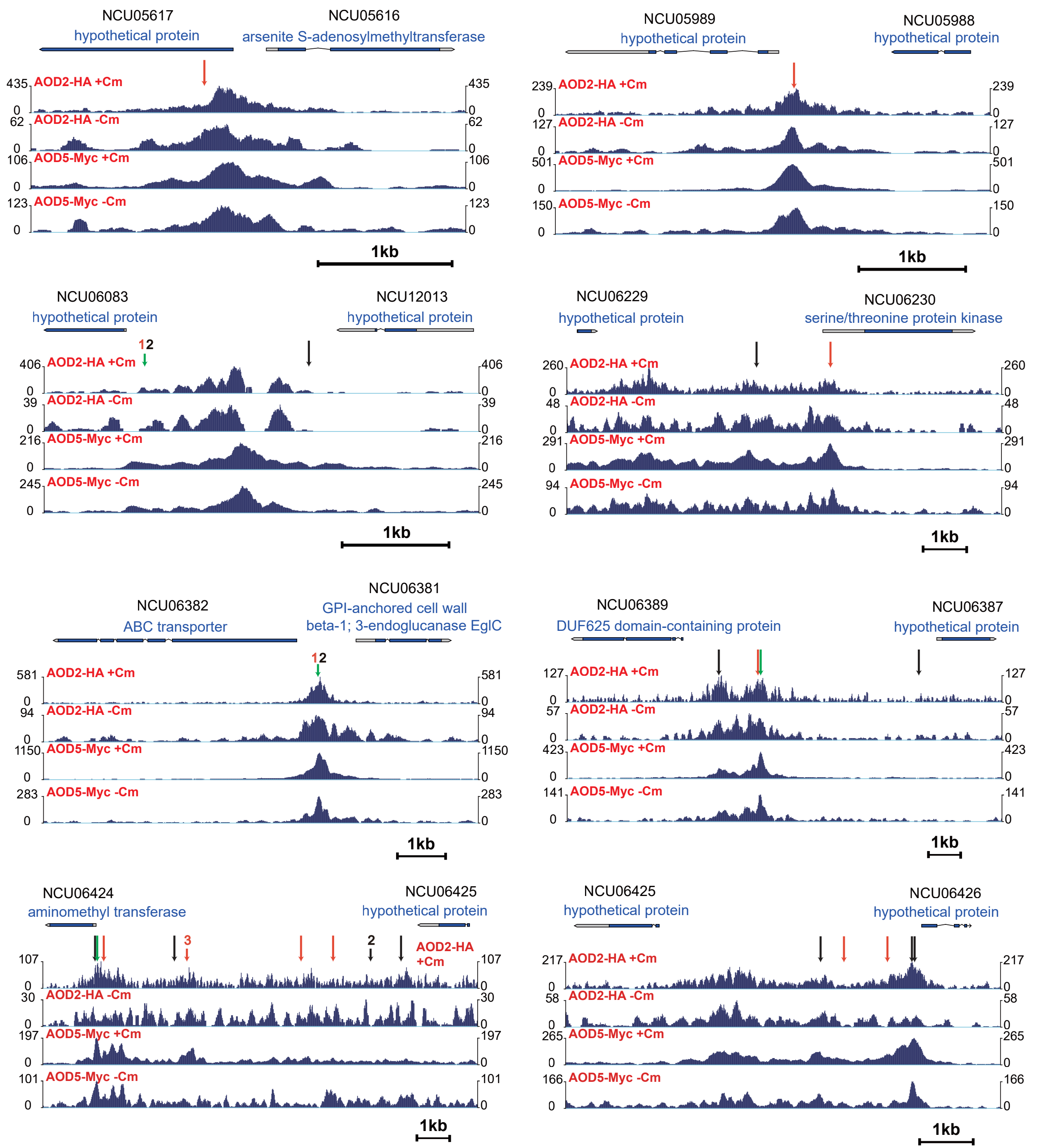

Figure S3 page 6

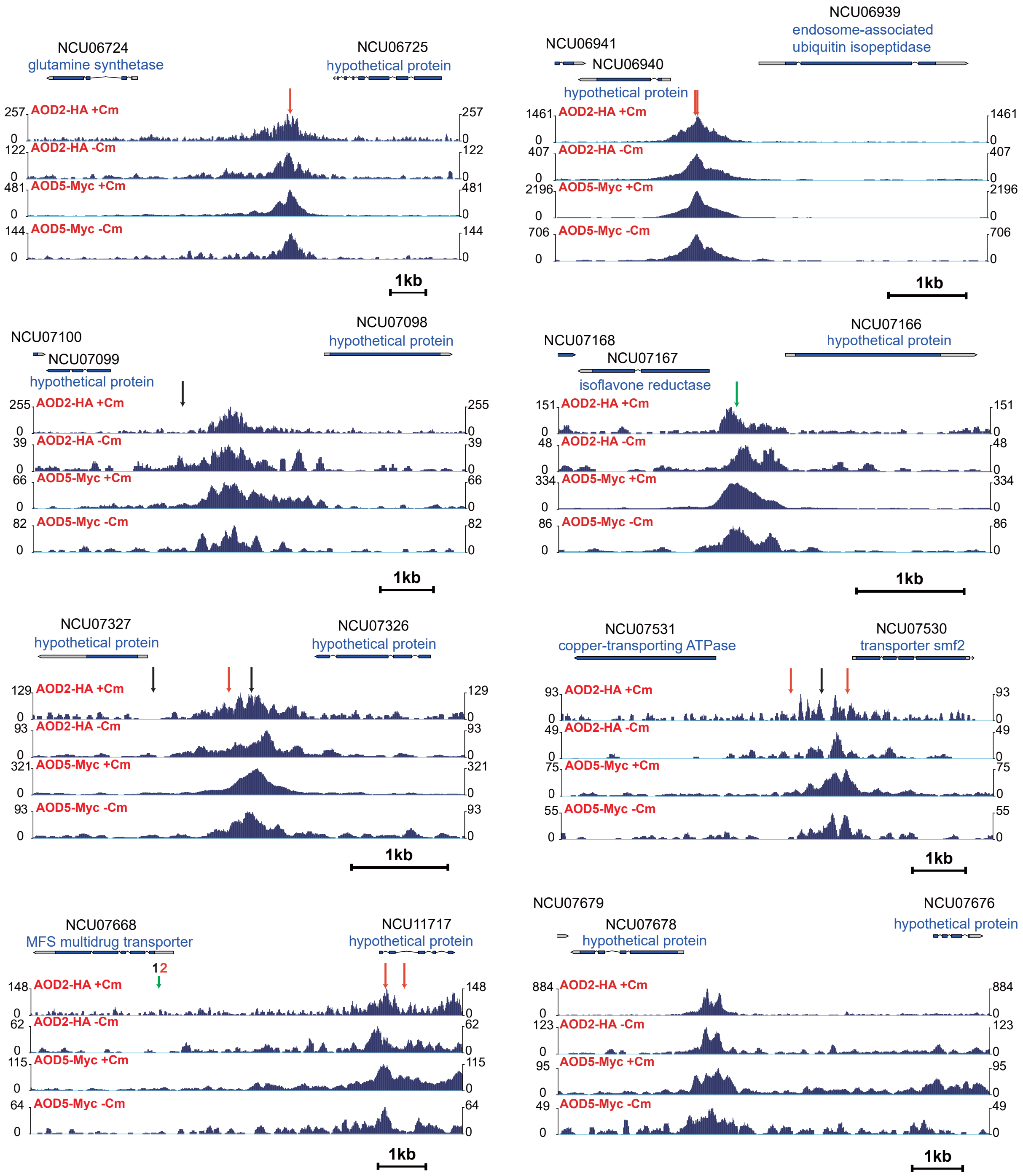

Figure S3 page 7

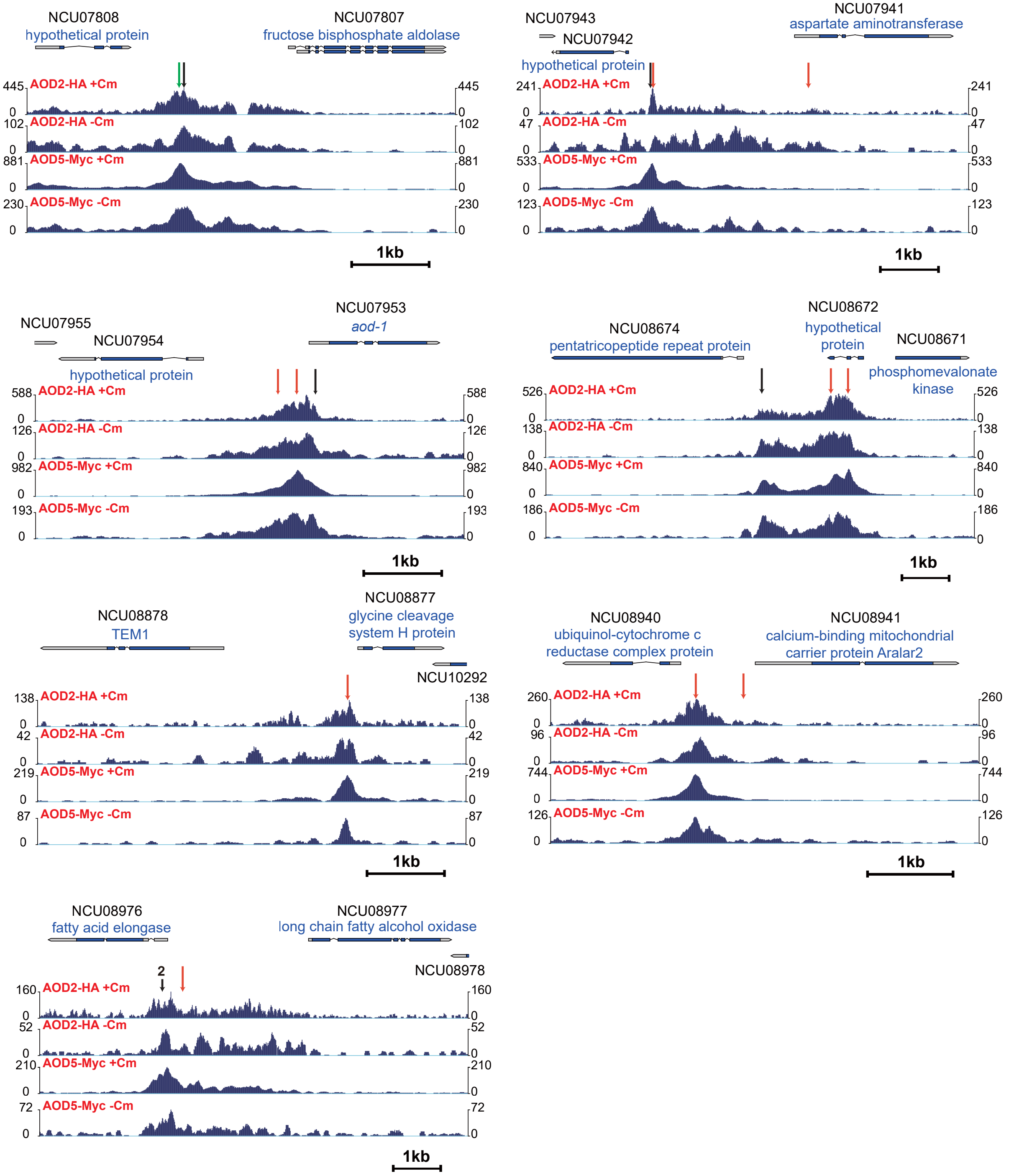

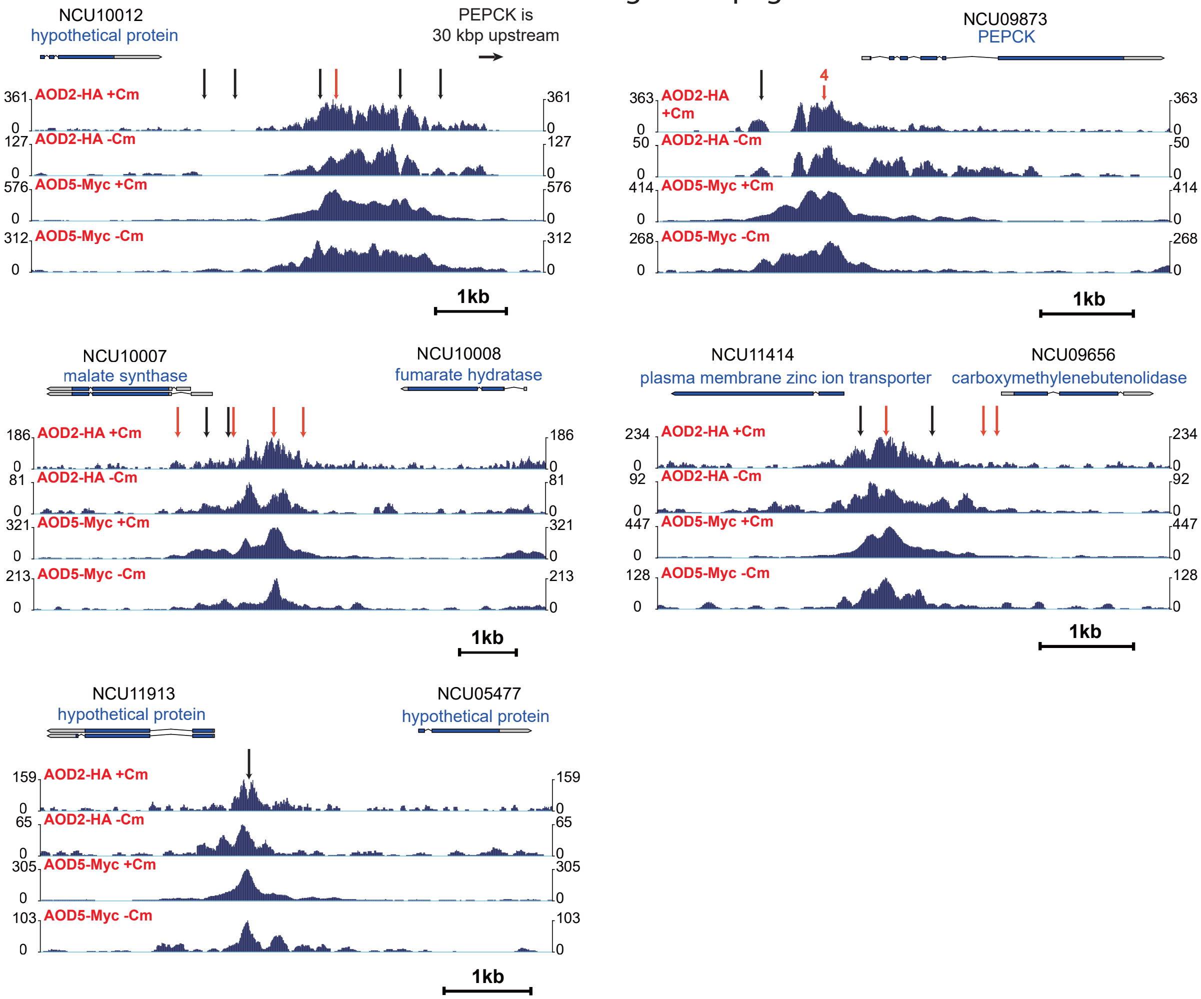

Figure S3. Position of AIM sequences in ChIP-seq peaks for the 65 peak sets in common to all four experiments. Peak sets from regions without an AIM sequence are not shown. Snapshots of the peak sets were taken from gbrowse. The y-axes were set for maximal visualization of the summits. The control peak sets are not shown. CGG(N7)CGG motifs on the + strand (DNA strand from 5' to 3' corresponds to the order from left to right on the gbrowse) are indicated by black arrows whereas CCG(N7)CCG motifs on the + strand are indicated by red arrows. In cases where CGG(N7)CGG and CCG(N7)CCG sequences are too close to each other to be resolved, green arrows are used. One green arrow is a mixture of a CGG(N7)CGG and a CCG(N7)CCG. Shorter green arrows with two numbers above indicate the number of CGG(N7)CGG sequences (black number) and CCG(N7)CCG sequences (red number). If there are two or more closely clustered CGG(N7)CGG repeats, the black arrows are shortened and the number on the top indicates the number of repeats. The same applies to CCG(N7)CCG repeats using shortened red arrows. The peaks at PEPCK (NCU09873) occur at two loci within a 35 kb intergenic region.
