# Supplementary material for: Alternative Oxidase Transcription Factors AOD2 and AOD5 of Neurospora crassa Control the Expression of Genes Involved in Energy Production and Metabolism
Source: G3 (Bethesda). 2016 Dec 16;7(2):449–66. doi: 10.1534/g3.116.035402 (PMC5295593; doi:10.1534/g3.116.035402)
Supplement: Supplementary file 8 [file 449FigureS5.docx]

Figure S5 page 1

**____________________________________________________________**

NCU02128 TTCCCACACTCAATCGCCTGTGCAGTCTTGGCGGACGTGTGTGTGTACACGCACACACGA 60

NCU01957 TTCCCACACTCAAACGCCTGTGCAGTCTTGGCGGACGTGTGCGTGTACACACACACACAA 60

NCU09144 TTCCCACACTCAAACGCCTGTGCAGTCTTGGCGGACGTGTGCGTGTACACACACACACAA 60

NCU03409 TTCCCACACTCAATCGCCTGTGCAGTCTTGGCGGACGTGTGTGTGTACACGCACACACGA 60

NCU04986 TTCCCACACTCAAACGCCTGTGCAGTCTTGGCGGACGTGTGCGTGTACACACACACACAA 60

NCU07676 TTCCCACACTCAATCGCCTGTGCAGTCTTGGCGGACGTGTGTGTGTACACGCACACACGA 60

NCU05202 TTCCCACACTCAAACGCCTGTGCAGTCTTGGCGGACGTGTGCGTGTACACACACACACAA 60

NCU04392 TTCCCACACTCAAACACCTGTGCAGTCTTGGCGGACGTGTGTGTGTACACACACACACAA 60

NCU01321 TTCCCACACTCAAACGCCTGTGCAGTCTTGGCGGACGTGTGCGTGTACACACACACACAA 60

NCU05527 TTCCCACACTCAAACGCCTGTGCAGTCTTGGCGGACGTGTGCGTGTACACACACACACAA 60

NCU09993 TTCCCACACTCAAACACCTGTGCAGTCTTGGCGGACGTATGTGTGTATATACACACACAA 60

************* * ********************** ** ***** * ******* *

**__________________**

NCU02128 CTTTTTGGCAAAACGGACTATCGATAAGATTTCGTTTGCTTATATCTCTACCAAACGAAG 120

NCU01957 CTTTTTGGCAAAACGGACCATCGATGAGATTTCATTTGCTTATATCTCTACCAAACAAAG 120

NCU09144 CTTTTTGGCAAAACGGACCATCGACAAAATTTCATTTGCTTATATCTCTACCAAACGAGG 120

NCU03409 CTTTTTGGCAAAACGGACTATCGATAAGATTTCGTTTGCTTATATCTCTACCAAACGAAG 120

NCU04986 CTTTTTGGCAAAACGGACCATCGACAAAATTTCATTTGCTTATATCTCTACCAAACGAGG 120

NCU07676 CTTTTTGGCAAAACGGACTATCGATAAGATTTCGTTTGCTTATATCTCTACCAAACGAAG 120

NCU05202 CTTTTTGGCAAAACGGACCATCGACAAAATTTCATTTGCTTATATCTCTACCAAACGAGG 120

NCU04392 CTTTTTGGCAAAATGGACCATCGATGAGATTTCATTTGCTTATATCTCTACCAAACGGGG 120

NCU01321 CTTTTTGGCAAAACGGACCATCGATGAGATTTCATTTGCTTATATCTCTACTAAACAAAG 120

NCU05527 CTTTTTGGCAAAACGGACCATCGATGAGATTTCATTTGCTTATATTTCTATTAAGCGAAG 120

NCU09993 CTTTTTGGCAAAATGGACTATCGATAAGATTTTATTTACTTATATCTCTACTAAACGGGG 120

************* **** ***** * **** *** ******* **** ** * *

NCU02128 AGATAAAAGAAAATGGACTCAAATTTTAGAATCGGCGTACCTAAGACTACTAAGACAATA 180

NCU01957 AGATAAAAGAAATCCGACTTGAGATTCAGAGTCAGCGTACTTAAGATTATTAAGAGAATA 180

NCU09144 AGATGAAAGAGAACCGACTTGAGATTTAGAATTAGAGTACTTAAAATTATCAAGAGAAAA 180

NCU03409 AGATAAAAGAAAATGGACTCAAATTTTAGAATCGGCGTACCTGGCGGAACTAAGACAATA 180

NCU04986 AGATGAAAGAGAACCGACTTGAGATTTAGAATTAGAGTACTTAAAATTATCAAGAGAAAA 180

NCU07676 AGATAAAAGAAAATGGACTCAAATTTTAGAATCGGCGTACCTAAGACTACTAAGACAATA 180

NCU05202 AGATGAAAGAGAACCGACTTGAGATTTAGAATTAGAGTACTTAAAATTATCAAGAGAAAA 180

NCU04392 AGATAGAGGAAAACCGACTTGAGATTTAGGATCAGCGTACTTAGAACTACTAAGAAA-TA 179

NCU01321 AGATAAAAGAAATCCGACTTGAGATTCAGAATTAGCGTACTTAGGATTGTTAAGAGAGAA 180

NCU05527 AGATAAAAGAAAATTGACTTGAAATTCAAAGTTAGTGTACTTAAGATTATCAAGAGACAA 180

NCU09993 AGATAAAGGAGAACCGACTTGAGATTTAGGATTAGCGTACTTAAAACTACTAAGAAAATA 180

**** * ** * **** * ** * * * **** * **** * *

**______**

NCU02128 ATCTTTT--CTATAGTATAATTGAATTTCTTCATCTTCGTCTTTCGTTTCGATGGTCCGT 238

NCU01957 GTTTTTTTTCCATAGGATAATTGAGTTTCTTCATCTTCGTCTTTCGTTTCAATAGTCCAT 240

NCU09144 GTTTTTT--CTATAGGATAATTGAATTCCTTCATCTTCGTCTTTCGTTTCAGTAGTCCAT 238

NCU03409 ATCTTTT--CTATAGTATAATTGAATTTCTTCATCTTCGTCTTTCGTTTCGATGGTCCGT 238

NCU04986 GTTTTTT--CTATAGGATAATTGAATTCCTTCATCTTCGTCTTTCGTTTCAGTAGTCCAT 238

NCU07676 ATCTTTT--CTATAGTATAATTGAATTTCTTCATCTTCGTCTTTCGTTTCGATGGTCCGT 238

NCU05202 GTTTTTT--CTATAGGATAATTGAATTCCTTCATCTTCGTCTTTCGTTTCAGTAGTCCAT 238

NCU04392 GTTTTTC--CTCCAGGATAATTACATCTCGTCATCTCCATCTTTCGTTTCAATGGTCCGT 237

NCU01321 GTTTTTT--CCATAGGATAATTGAATTTATTCATCTTCGTCTTTCGTTTCAATAGTCCAT 238

NCU05527 GTTTTTT--CTATAGGATAATTGAATTTCTTCATCTTCGTCTTTCGTTTCAATAGTCCAT 238

NCU09993 GTTTTTC--CTCTAGGATAATTGCATTTCGTCATCTCCATCTTTCGTTTCGATAGTCCGT 238

* *** * ** ****** * ****** * *********** * **** *

Figure S5 page 2

**____________________________________________________________**

NCU02128 TTTGCCAAAAAGTCGTGTGTGCGTGTACACACACACATCCGCCAAGACTGCACAGGCGAT 298

NCU01957 TTTACCAAAAAGTTGTGTGTGTGTGTACACGCACACGTCCGCCAAGACTGCACAGGCGTT 300

NCU09144 TTTGCCAAAAAGTTGTGTGTGTGTGTACACGCACACGTCCGCCAAGACTGCACAGGCGTT 298

NCU03409 TTTGCCAAAAAGTCGTGTGTGCGTGTACACACACACATCCGCCAAGACTGCACAGGCGAT 298

NCU04986 TTTGCCAAAAAGTTGTGTGTGTGTGTACACGCACACGTCCGCCAAGACTGCACAGGCGTT 298

NCU07676 TTTGCCAAAAAGTCGTGTGTGCGTGTACACACACACATCCGCCAAGACTGCACAGGCGAT 298

NCU05202 TTTGCCAAAAAGTTGTGTGTGTGTGTACACGCACACGTCCGCCAAGACTGCACAGGCGTT 298

NCU04392 TTTGCCAAAAAGTTGTGTGTGTGTGTACACGCACACGTCCGCCAAGACTGCACAGGTGTT 297

NCU01321 TTTACCAAAAAGTTGTGTGTGTGTGTACACGCACACGTCCGCCAAGACTGCACAGGCGTT 298

NCU05527 TTTGCCAAAAAGTTGTGTGTGTGTGTACACGCACACGTCCGCCAAGACTGCACAGGCGTT 298

NCU09993 TTTGCCAAAAAGTTGTGTGTGTGTGTACACGCACACGTCCGCCAAGACTGCACAGGTGTT 298

*** ********* ******* ******** ***** ******************* * *

**____________**

NCU02128 TGAGTGTGGGAA 310

NCU01957 TGAGTGTGGGAA 312

NCU09144 TGAGTGTGGGAA 310

NCU03409 TGAGTGTGGGAA 310

NCU04986 TGAGTGTGGGAA 310

NCU07676 TGAGTGTGGGAA 310

NCU05202 TGAGTGTGGGAA 310

NCU04392 TGAGTGTGGGAA 309

NCU01321 TGAGTGTGGGAA 310

NCU05527 TGAGTGTGGGAA 310

NCU09993 TGAGTGTGGGAA 310

************

**Figure S5. Alignment of repeat regions.** The 11 repeat regions found to be greater than 75% identical to the repeat near NCU02128 are aligned in the order in which they appear in Table 5. Identical bases in all eleven sequences are indicated by an asterisk below the alignment. The 78 base pair inverted repeat regions are indicated by a red overline. The 14 base pair consensus sequence found at the summit of the AOD2 binding sites are highlighted in yellow. All the repeat sequences shown display AOD2 binding except the final sequence associated with NCU09993.
