# Supplementary material for: Alternative Oxidase Transcription Factors AOD2 and AOD5 of Neurospora crassa Control the Expression of Genes Involved in Energy Production and Metabolism
Source: G3 (Bethesda). 2016 Dec 16;7(2):449–66. doi: 10.1534/g3.116.035402 (PMC5295593; doi:10.1534/g3.116.035402)
Supplement: Supplementary file 9 [file 449FigureS6.pdf]

Figure S6 page 1

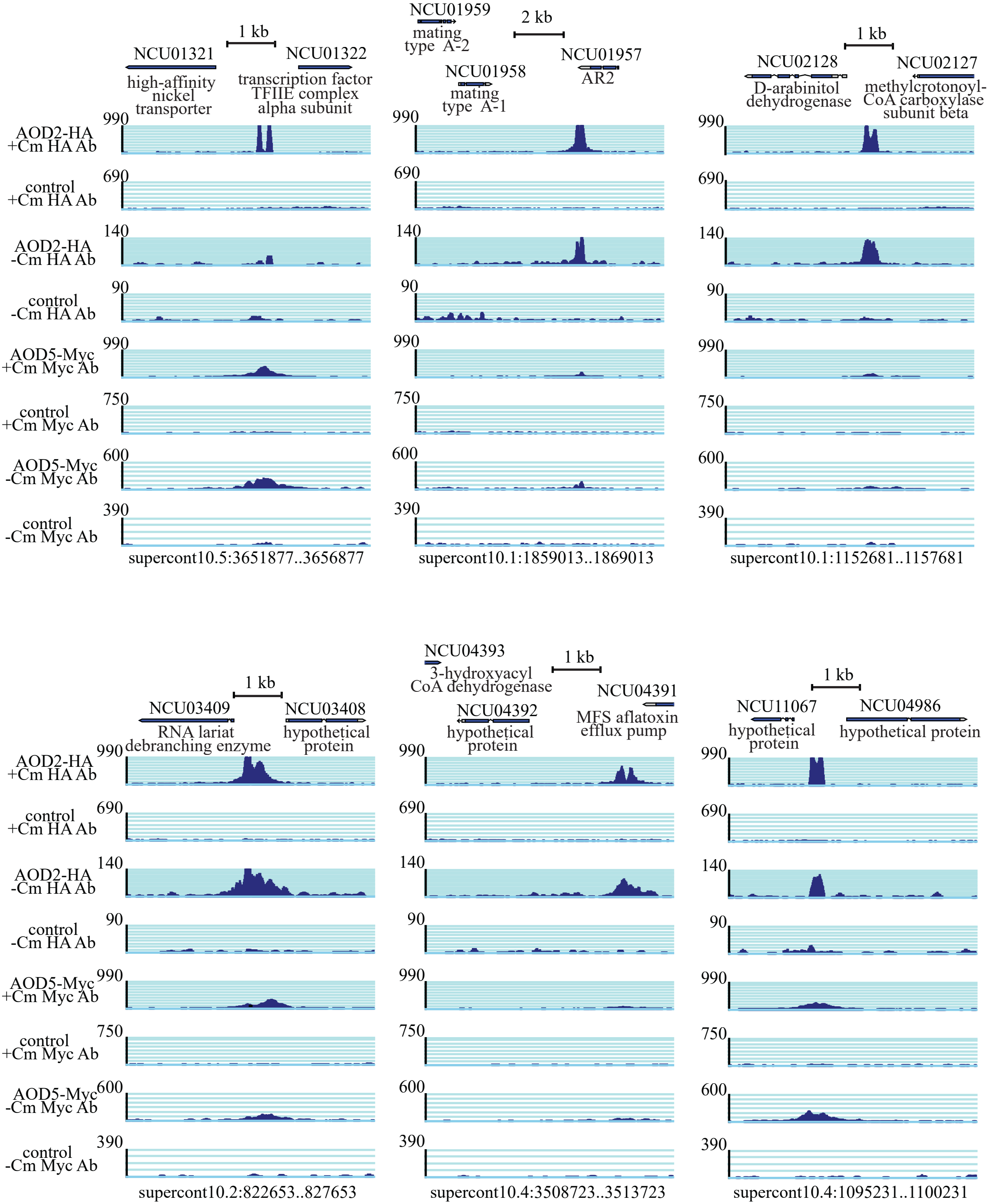

Figure S6 page 2

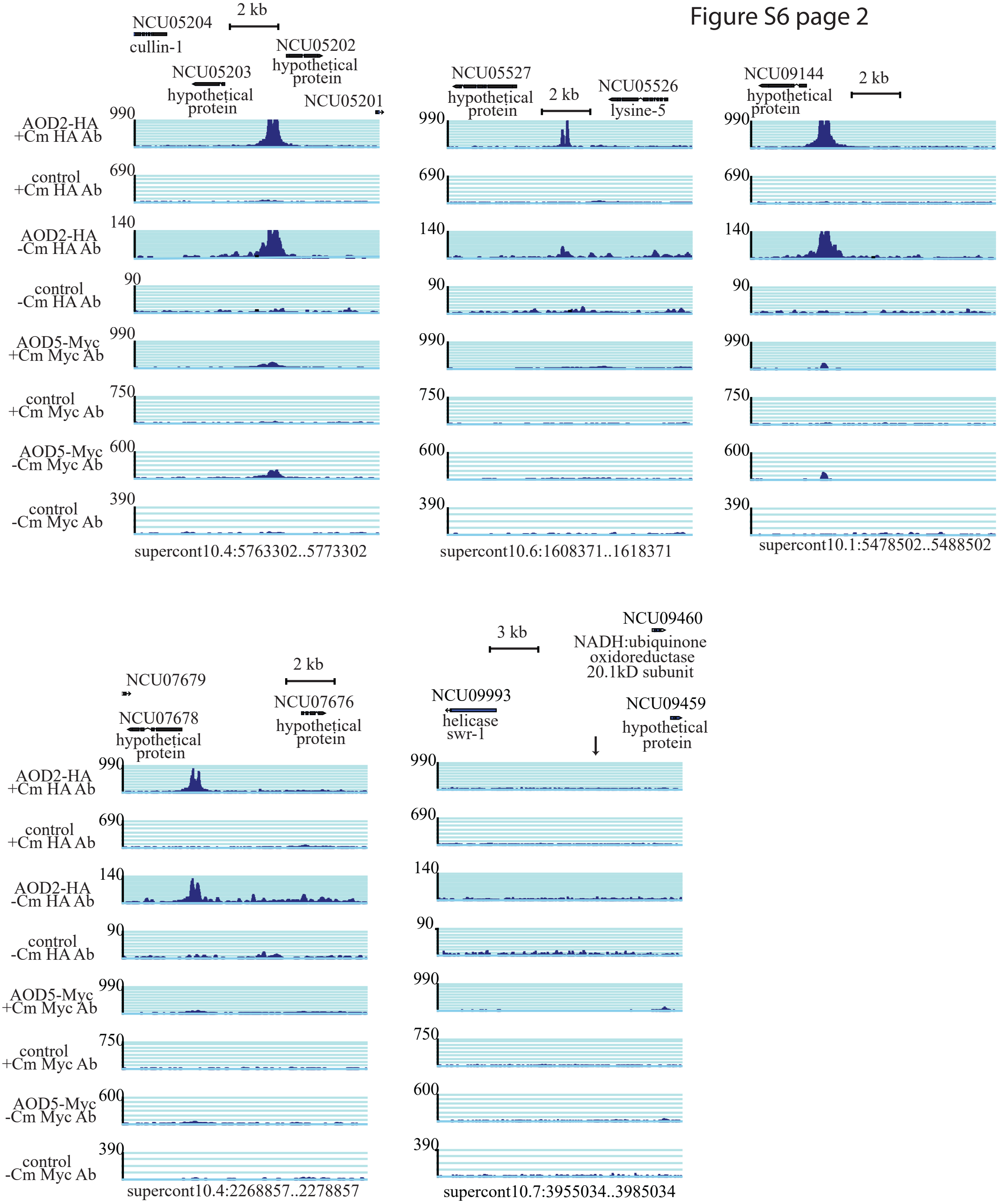

Figure S6. Peaks of AOD2 and AOD5 binding at the 11 repeat sequence regions. Snapshots from gbrowse are shown. The ratios of the y-axis in different immunoprecipitations were assigned based on the number of mapped sequence reads obtained in the individual experiments for a given antibody and are the same as those on Figure 5. The position of genes in each region is shown at the top. The individual experiments are listed on the left with respect to the tagged protein in the strain used, the growth condition (+ or – Cm), and the antibody (Ab) used for immunoprecipitation. The control ChIP-seq experiments were performed on strain NCN251 (expresses no HA- or myc-tagged proteins), exactly as with the strains expressing tagged proteins. In the last panel, the arrow shows the position of the repeat sequence, where no binding is evident.
