# Supplementary material for: Alternative Oxidase Transcription Factors AOD2 and AOD5 of Neurospora crassa Control the Expression of Genes Involved in Energy Production and Metabolism
Source: G3 (Bethesda). 2016 Dec 16;7(2):449–66. doi: 10.1534/g3.116.035402 (PMC5295593; doi:10.1534/g3.116.035402)
Supplement: Supplementary file 11 [file 449FigureS7.docx]

Figure S7. Sites where AOD2 binding exceeds AOD5 binding that are not associated with repeat regions. (.ai, 1.29 MB)

<http://www.g3journal.org/lookup/suppl/doi:10.1534/g3.116.035402/-/DC1/FigureS7.ai>
