# Supplementary material for: Alternative Oxidase Transcription Factors AOD2 and AOD5 of Neurospora crassa Control the Expression of Genes Involved in Energy Production and Metabolism
Source: G3 (Bethesda). 2016 Dec 16;7(2):449–66. doi: 10.1534/g3.116.035402 (PMC5295593; doi:10.1534/g3.116.035402)
Supplement: Supplementary file 12 [file 449TableS1.docx]

**Table S1. Genes identified as overrepresented by fungifun2 analysis of the 98 genes in Table 3. A total of 23 different genes were identified but several of these are present in more than one FunCat description category as shown in the Table.**

| Gene | FunCat ID | FunCat description | FunCat main category | Exact p-value | Adjusted p-value |
| --- | --- | --- | --- | --- | --- |
| NCU00865  (oxalate decarboxylase) | 2.13 | respiration | Energy | 0.00020557 | 0.0078115 |
| NCU01195  (gdh) | 01.01.03.02.01 | biosynthesis of glutamate | Metabolism | 0.000014856 | 0.0022581 |
| NCU01744  (glutamate synthase) | 01.01.03.02.01 | biosynthesis of glutamate | Metabolism | 0.000014856 | 0.0022581 |
| NCU01744 | 01.01.03 | assimilation of ammonia, metabolism of the glutamate group | Metabolism | 0.001087 | 0.024929 |
| NCU01808  (cytochrome c) | 02.13.03 | aerobic respiration | Energy | 0.000032444 | 0.0024658 |
| NCU01808 | 2.13 | respiration | Energy | 0.00020557 | 0.0078115 |
| NCU01808 | 2.11 | electron transport and membrane-associated energy conservation | Energy | 0.00044414 | 0.013502 |
| NCU01808 | 20.03 | transport facilities | Cellular transport, transport facilitation and transport routes | 0.001148 | 0.024929 |
| NCU02475  (glycine dehydrogenase) | 01.01.09.01.02 | degradation of glycine | Metabolism | 0.000084618 | 0.0042873 |
| NCU02514  (ATPase-1) | 02.13.03 | aerobic respiration | Energy | 0.000032444 | 0.0024658 |
| NCU02514 | 2.13 | respiration | Energy | 0.00020557 | 0.0078115 |
| NCU02514 | 2.11 | electron transport and membrane-associated energy conservation | Energy | 0.00044414 | 0.013502 |
| NCU02549  (processing enhancing protease) | 02.13.03 | aerobic respiration | Energy | 0.000032444 | 0.0024658 |
| NCU02549 | 2.11 | electron transport and membrane-associated energy conservation | Energy | 0.00044414 | 0.013502 |
| NCU03257  (ammonium transporter MEP1) | 20.03 | transport facilities | Cellular transport, transport facilitation and transport routes | 0.001148 | 0.024929 |
| NCU04569  (5-oxoprolinase) | 01.01.03.02.01 | biosynthesis of glutamate | Metabolism | 0.000014856 | 0.0022581 |
| NCU04569 | 01.01.03 | assimilation of ammonia, metabolism of the glutamate group | Metabolism | 0.001087 | 0.024929 |
| NCU04874  (aod-3) | 02.13.03 | aerobic respiration | Energy | 0.000032444 | 0.0024658 |
| NCU04874 | 2.13 | respiration | Energy | 0.00020557 | 0.0078115 |
| NCU05299  (NADH-ubiquinone reductase 29.9) | 2.13 | respiration | Energy | 0.00020557 | 0.0078115 |
| NCU05299 | 2.11 | electron transport and membrane-associated energy conservation | Energy | 0.00044414 | 0.013502 |
| NCU05390  (mt phosphate carrier) | 20.03 | transport facilities | Cellular transport, transport facilitation and transport routes | 0.001148 | 0.024929 |
| NCU06382  (ABC transporter) | 20.03 | transport facilities | Cellular transport, transport facilitation and transport routes | 0.001148 | 0.024929 |
| NCU06424  (amino methyl transferase) | 01.01.09.01.02 | degradation of glycine | Metabolism | 0.000084618 | 0.0042873 |
| NCU06724  (glutamine synthase) | 01.01.03.02.01 | biosynthesis of glutamate | Metabolism | 0.000014856 | 0.0022581 |
| NCU06724 | 01.01.03 | assimilation of ammonia, metabolism of the glutamate group | Metabolism | 0.001087 | 0.024929 |
| NCU07531  (Cu transporting ATPase) | 20.03 | transport facilities | Cellular transport, transport facilitation and transport routes | 0.001148 | 0.024929 |
| NCU07668  (MFS multidrug transporter) | 20.03 | transport facilities | Cellular transport, transport facilitation and transport routes | 0.001148 | 0.024929 |
| NCU07941  (aspartate amino transferase) | 01.01.03.02.01 | biosynthesis of glutamate | Metabolism | 0.000014856 | 0.0022581 |
| NCU07953  (aod-1) | 02.13.03 | aerobic respiration | Energy | 0.000032444 | 0.0024658 |
| NCU07953 | 2.13 | respiration | Energy | 0.00020557 | 0.0078115 |
| NCU08877  (glycine cleavage system H) | 01.01.09.01.02 | degradation of glycine | Metabolism | 0.000084618 | 0.0042873 |
| NCU08940  (complex III protein) | 02.13.03 | aerobic respiration | Energy | 0.000032444 | 0.0024658 |
| NCU08940 | 2.11 | electron transport and membrane-associated energy conservation | Energy | 0.00044414 | 0.013502 |
| NCU08940 | 20.03 | transport facilities | Cellular transport, transport facilitation and transport routes | 0.001148 | 0.024929 |
| NCU08941  (Ca binding mt carrier) | 20.03 | transport facilities | Cellular transport, transport facilitation and transport routes | 0.001148 | 0.024929 |
| NCU08947  (complex III protein) | 02.13.03 | aerobic respiration | Energy | 0.000032444 | 0.0024658 |
| NCU08947 | 2.11 | electron transport and membrane-associated energy conservation | Energy | 0.00044414 | 0.013502 |
| NCU08947 | 20.03 | transport facilities | Cellular transport, transport facilitation and transport routes | 0.001148 | 0.024929 |
