# Supplementary material for: Alternative Oxidase Transcription Factors AOD2 and AOD5 of Neurospora crassa Control the Expression of Genes Involved in Energy Production and Metabolism
Source: G3 (Bethesda). 2016 Dec 16;7(2):449–66. doi: 10.1534/g3.116.035402 (PMC5295593; doi:10.1534/g3.116.035402)
Supplement: Supplementary file 15 [file 449FileS2.docx]

File S2. Excel table showing the results of MACS2 analysis of ChIP-seq data from the HA tagged AOD2 strain grown in the absence of Cm (FN4) following subtraction of control data (FN5). See Table 2 for experimental details and number of mapped reads. (.xlsx, 136 KB)

[http://www.g3journal.org/lookup/suppl/doi:10.1534/g3.116.035402/-/DC1/FileS2.xlsx](http://www.g3journal.org/lookup/suppl/doi:10.1534/g3.116.035402/-/DC1/FileS1.xlsx)
