# Supplementary material for: Alternative Oxidase Transcription Factors AOD2 and AOD5 of Neurospora crassa Control the Expression of Genes Involved in Energy Production and Metabolism
Source: G3 (Bethesda). 2016 Dec 16;7(2):449–66. doi: 10.1534/g3.116.035402 (PMC5295593; doi:10.1534/g3.116.035402)
Supplement: Supplementary file 19 [file 449FileS4.docx]

File S4. Excel table showing the results of MACS2 analysis of ChIP-seq data from the Myc tagged AOD5 strain grown in the absence of Cm (FN6) following subtraction of control data (FN7). See Table 2 for experimental details and number of mapped reads. (.xlsx, 84 KB)

<http://www.g3journal.org/lookup/suppl/doi:10.1534/g3.116.035402/-/DC1/FileS4.xlsx>
